# A reference high-density genetic map of greater yam (*Dioscorea alata* L.)

**DOI:** 10.1007/s00122-019-03311-6

**Published:** 2019-02-20

**Authors:** Fabien Cormier, Floriane Lawac, Erick Maledon, Marie-Claire Gravillon, Elie Nudol, Pierre Mournet, Hélène Vignes, Hâna Chaïr, Gemma Arnau

**Affiliations:** 1 CIRAD, UMR AGAP, 97170 Petit-Bourg, Guadeloupe, France; 2 Univ. Montpellier, CIRAD, INRA, Montpellier SupAgro, Montpellier, France; 3 VARTC, P.O. Box 231, Luganville, Santo, Vanuatu; 4 CIRAD, UMR AGAP, 34398 Montpellier, France

## Abstract

**Key message:**

**This study generated the first high-density genetic map for** ***D. alata*** **based on genotyping-by-sequencing and provides new insight on sex determination in yam.**

**Abstract:**

Greater yam (*Dioscorea alata* L.) is a major staple food in tropical and subtropical areas. This study aimed to produce the first reference genetic map of this dioecious species using genotyping-by-sequencing. In this high-density map combining information of two F1 outcrossed populations, 20 linkage groups were resolved as expected and 1579 polymorphic markers were ordered. The consensus map length was 2613.5 cM with an average SNP interval of 1.68 cM. An XX/XY sex determination system was identified on LG6 via the study of sex ratio, homology of parental linkage groups and the identification of a major QTL for sex determination. Homology with the sequenced *D. rotundata* is described, and the median physical distance between SNPs was estimated at 139.1 kb. The effects of segregation distortion and the presence of heteromorphic sex chromosomes are discussed. This *D. alata* linkage map associated with the available genomic resources will facilitate quantitative trait mapping, marker-assisted selection and evolutionary studies in the important yet scarcely studied yam species.

**Electronic supplementary material:**

The online version of this article (10.1007/s00122-019-03311-6) contains supplementary material, which is available to authorized users.

## Introduction

Yams (*Dioscorea* spp.) are important food security crops that are grown in tropical and subtropical regions (Coursey 1967). They are dioecious herbaceous vines cultivated for their starchy tubers with a high nutritional content (Muzac-Tucker et al. 1993). *D. rotundata* and *D. alata* are the two main cultivated species *(*Ayensu and Coursey 1972) and belong to the same botanical section, i.e., Enantiophyllum (Wilkin et al. 2005), which is one of the latest diverging lineages in *Discorea* (Viruel et al. 2016).

Greater yam (*D. alata* L.) also named water or winged yam ranks second in production, and it is the most widely distributed yam species in the world (Abraham and Gopinathan Nair 1990). It is a strictly dioecious and polyploid species (2*n *= 40, 60, 80) with a basic chromosome number of 20 (Arnau et al. 2009). Diversity studies have shown that the most common forms are diploids, followed by triploids, and that tetraploids are rare (Arnau et al. 2017). It is superior to most cultivated yam species in terms of yield potential (especially under low soil fertility), ease of propagation, competition with weeds (early vigor) and tuber storability (Sartie and Asiedu 2014). Consequently, the importance of *D*. *alata* in terms of food security has given rise to several genetic improvement programs throughout tropical regions which are aimed at developing new varieties with high yield, tuber quality and resistance to pests and diseases (Abraham and Gopinathan Nair 1990; Egesi and Asiedu 2002; Arnau et al. 2011) such as anthracnose, caused by *Colletotrichum gloeosporioides* (Abang et al. 2004). Breeding of this heterozygous crop is essentially carried out on the basis of phenotypic observations and is a long and difficult process.

One prerequisite for the development of marker-assisted breeding tools is the development of linkage analysis or/and association mapping. Both approaches are based on ordered genetic information. However, the *D. alata* genome has yet to be completely sequenced, and it is only available as unordered scaffolds (*D. alata* genome assembly Version 1, Water Yam Genome Project, ftp://yambase.org/genomes/Dioscorea_alata/). Two linkage maps were constructed using dominant amplified fragment length polymorphism markers (AFLP; Mignouna et al. 2002; Petro et al. 2011).

Codominant markers such as microsatellites (simple sequence repeats, SSRs) and single nucleotide polymorphisms (SNPs) are choice markers for plant breeding applications as they allow estimation of additive and dominant allelic effects. SSR markers have been generated in *D. alata* and other *Dioscorea* spp. (Terauchi and Konuma 1994; Misuki et al. 2005; Tostain et al. 2006; Hochu et al. 2006; Andris et al. 2010; Saski et al. 2015). Moreover, an EST-SSR genetic linkage map containing 380 markers was recently published (Bhattacharjee et al. 2018). Thus, the marker number is still limited for genome-wide approaches necessary for association genetics, and their implementation cost is relatively high.

With the development of next-generation sequencing (NGS) methods, SNPs are now the most widely available markers for high-throughput genotyping. Genotyping-by-sequencing (GBS) allows the detection and genotyping of tens of thousands of SNPs in many individuals (DePristo et al. 2011; Davey et al. 2011; Elshire et al. 2011), resulting in an unparalleled cost per data point when screening for codominant polymorphisms in large panels and for constructing highly saturated genetic maps (Poland et al. 2012, Ward et al. 2013).

The objective of the present study was to overcome the main limitations in identifying genomic regions linked to agronomic traits of interest in *D. alata* by establishing a high-density genetic map of *D. alata* using genotyping-by-sequencing. The relationship between genetic mapping and sex determination was also investigated since dioecy can be related to the chromosome architecture (Kumar et al. 2014).

## Materials and methods

### Materials

The mapping populations consisted of two greater yam (*Dioscorea alata*) full-sib F1 segregating populations. The hybridizations were performed in the French West Indies (Roujol, Petit-Bourg, Guadeloupe) using diploid parents (flow cytometry; Arnau et al. 2009). Population A was derived from a cross between a female breeding line (74F) developed at the French Agricultural Research Centre for International Development (CIRAD) and a male Caribbean landrace (Kabusa). Population B was derived from a cross between the same female (74F) and another male breeding line developed at CIRAD (14 M).

Overall, 250 and 360 pollinations were manually carried out for population A and population B, respectively. Once harvested (60–70 days after pollination), the fruits were left to soak in 70% isopropyl alcohol and then in 12% sodium hypochlorite for 5 min in each solution before rinsing with distilled water. Embryo rescue procedures were performed to conserve clean material and speed up multiplication. A total of 140 and 280 individuals were micropropagated for population A and population B, respectively. All progenies and parents were then transferred to an experimental field in two complete blocks with nine repetitions.

### DNA extraction and genotyping-by-sequencing (GBS)

Young leaves from the same vine for each progeny and parent were collected, stored in coffee filters and then dried at 45 °C overnight. Total genomic DNA extractions were performed from dried leaves by an automated method adapted from Risterucci et al. (2009) on Biomek FXP (Beckman Coulter, CA, USA) and using the NucleoMag Plant Kit (Macherey–Nagel, Germany). DNA samples were quantified with a Fluoroskan Ascent FL fluorometer (Thermo Fisher Scientific, Waltham, MA, USA). Genomic DNA quality was checked using agarose gel electrophoresis. A genomic library was prepared using PstI-MseI (New England Biolabs, Hitchin, UK) restriction enzymes with a normalized 200 ng quantity of DNA per sample. The procedures published by Elshire et al. (2011) were followed; however, the common adapter was replaced to be complementary to MseI recognition site. Digestion and ligation reactions were conducted in the same plate. Digestion was conducted at 37 °C for 2 h and then at 65 °C for 20 min to inactivate the enzymes. The ligation reaction was done using T4 DNA ligase enzymes (New England Biolabs, Hitchin, UK) at 22 °C for 1 h, and the ligase was then inactivated by heating at 65 °C for 20 min. Parents were replicated twice per plate. Ligated samples were pooled and PCR-amplified (18 cycles). The PCR-amplified libraries were purified using the Wizard PCR preps DNA purification system Promega (Madison, USA) and verified with the Agilent D5000 ScreenTape (Santa Clara, USA). Single-end sequencing of 150 base-pair reads was performed in a single lane on an Illumina HiSeq 3000 system (at the GeT-PlaGe platform in Toulouse, France). Losses during micropropagation and transfer to the field led to the genotyping-by-sequencing of 121 progenies for population A (74F × Kabusa) and 193 for population B (74F × 14 M). Parents were replicated to ensure SNP detection and high-quality parental information for the estimation of marker segregation types.

### SNP calling and filtering

Raw sequencing data were demultiplexed with GBSX v1.2 (Herten et al. 2015). Cutadapt v1.9 (Martin 2011) was used to trim adapters (options: -a AGATCGGAAGAGCG -O 10 -q 20,20 -m 30). SNP calling was done using the process_reseq. 1.0.py (python2) program followed by site pre-filtering using the VcfPreFilter.1.0.py (python2) program with the default parameters. Both programs are part of the VcfHunter package (Garsmeur et al. 2018; available at https://github.com/SouthGreenPlatform/VcfHunter/). As there was no complete *D. alata* reference genome, reads were aligned to the *D. rotundata* reference genome (pseudo-chromosomes BDMI0100001-21; Tamiru et al. 2017). SNPs were thus named according to their position in this *D. rotundata* reference genome.

SNPs and progenies were filtered by population using the following filters: minimum depth 10, maximum depth 500, missing data per site < 20%, missing data per individual < 40%, minor allele frequencies per site > 10% and minimum count read for heterozygous genotype > 3. Sites with missing data on parents or segregation patterns in progenies that were not in agreement with the parental genotypes were also excluded. Dataset filtering and formatting were coded in R 3.4.4 (R core team 2017) and based on the vcfR 1.5.0 package (Knaus and Grünwald 2017) to import vcf files into the R environment.

### Linkage analysis and parental map construction

SNPs with 1:1 Mendelian segregation if segregating only in one parent and 1:2:1 if segregating in both parents were retained for linkage analysis. SNPs with a significant segregation deviation within families (χ^2^ test; *P* < 0.001) were eliminated. Finally, SNPs were thinned to maintain a minimum spacing of 100 bp between adjacent markers.

Linkage analysis and map constructions were conducted separately for each parent by population according to the cross-pollinated (CP) model in JoinMap 4.1 software (Van Ooijen 2012). In each family, the “hk × hk” segregation patterns were used for both parents, while “lm × ll” and “nn × np” segregation patterns were used for the female and male parental maps, respectively. Regarding the difference in dataset size, linkage groups were established using a grouping LOD threshold value of 7 for the parents of population A and 5 for those of population B. Parental maps were computed using recombination frequencies below 0.45, LODs over 1.0, a regression algorithm with two ordering rounds and the Kosambi mapping function.

### QTL detection for sex determination

Sex was determined by looking at the type of inflorescence produced by each progeny. Phenotyping was conducted in 2016 and 2017 in both blocks to deal with the erratic flowering of *D. alata* and to avoid vine mixing issues. Overall, 69 progenies (32 females and 37 males) in population A (74F × Kabusa) and 75 progenies (31 females and 44 males) in population B (74F × 14 M) were phenotyped with confidence.

QTL detection was conducted on the four parental genetic maps using the R/qtl 1.42-8 package (Broman et al. 2003) and a simple interval mapping approach (options: step = 1 cM, error.prob = 1e−08, map.function = “kosambi”, model = “binary”, method = “hk”). Significance thresholds were calculated through permutations (1000) with an alpha risk of 0.05. QTL confidence intervals were computed using the “bayesint” function and 0.95 probability coverage of the interval.

### Construction of the *D. alata* reference map and comparison with *D. rotundata*

As the two mapping populations were derived from crosses involving the same female (74F), an integrated map of the female parent (74F) was computed using the JoinMap “combine groups for map integration” function. Three genetic maps of female 74F were thus generated: from the population A dataset (74F_A), the population B dataset (74F_B) and the integrated map (74F). The final *D. alata* consensus map was constructed using this same function and starting from the integrated female linkage groups (74F) and both male parent groups (Kabusa and 14 M). Homology between the four parental maps, the integrated female map and the final consensus map were visualized using the R package ggplot2 2.1.1 (Wickham 2016).

The *D. alata* genome assembly v1 available as scaffolds accounting for roughly half of the genome and 80–90% of protein-coding loci (Water Yam Genome Project—ftp://yambase.org/genomes/Dioscorea_alata/) was anchored to our consensus map. To do that, SNP flanking sequences (60 bp upstream and 60 bp downstream around the variant position) were extracted using SNiPlay3 (Dereeper et al. 2015). These sequences were mapped on *D. alata* scaffolds using BLAST (Basic Local Alignment Search Tool, ncbi-blast v2.2.30).The results were parsed using an E-value threshold of 1e-10 and keeping secondary hits only if the difference [− log10(best hit E-value)] − [− log10(hit E-value)] was lower than 2.

The consensus map was also compared to the *D. rotundata* reference genome (pseudo-chromosomes BDMI0100001-21; Tamiru et al. 2017). The *D. rotundata* genome was divided into pieces cutting halfway between SNPs included in our *D. alata* reference genetic map. The resulting genomic fragments were then reordered according to the SNP positions in the *D. alata* reference map developed in this study. Synteny between *D. alata* and *D. rotundata* was visualized using a Circos approach via the circlize R package 0.4.3 (Gu 2014).

## Results

### Genotyping-by-sequencing and SNP filtering

Overall, 121 progenies from population A (74F × Kabusa) and 193 from population B (74F × 14 M) were genotyped. Around 4.4 and 3.6 million reads per progeny were obtained with 83.9% and 82.5% of the reads mapping on the *D. rotundata* genome used as reference sequence, and for population A (74F × Kabusa) and population B (74F × 14 M), respectively (Online Resource 1). For the female (74F), 25 million reads were obtained, 84.6% of which were mapped. Nineteen million and 21 million reads were obtained, 82.2% and 84.2% of which were mapped for Kabusa and 14 M male parents, respectively. On average, 10% of the mapped reads were aligned to multiple positions (Online Resource 1) and removed.

By population, SNP filtering on the genotypic information quality (i.e., depth and allele frequencies) resulted in the detection of 29,224 and 11,808 SNPs in populations A and B, respectively (Table 1). By excluding sites based on missing data or discrepancies between segregation patterns in progenies and parental genotypes, 17,446 and 5434 SNPs were conserved for populations A and B, respectively. The segregation distortion threshold discarded 33% of SNPs in population A and 59% in population B (Table 1). Keeping a maximum of one SNP every 100 bp reduced the SNP dataset by approximately half. Thus, 5373 SNPs and 1075 SNPs were used for linkage analysis in population A and population B, respectively. Because of the missing data threshold per progeny, the final dataset included 79 progenies for population A and 110 progenies for population B.

**Table 1:** Summary of SNP filtering and dataset sizes per population

Population	High-quality SNPs	Low missing data and adequate segregation pattern	Undistorted SNPs	Dataset used in linkage mapping				
				No. of SNPs	No. of progenies	SNP depth^a^	NA per site^a^ (%)	NA per progeny^a^ (%)
A	29,224	17,446	11,667	5373	79	113	12.2	7.5
B	11,808	5434	2227	1075	110	88	14.2	8.6

The SNPs used in linkage mapping are the undistorted SNPs (χ^2^ test; *P* < 0.001) thinned so that no two sites were within 100 base pairs

^a^Median

### Linkage analysis and parental maps

A total of 5837 unique SNPs were used in the linkage analysis, with 611 SNPs being common to both populations. Although a single female parent (74F) was used, common SNPs were not homogeneously distributed across segregation patterns between populations (Table 2). The proportions of markers segregating only in the female or male parent were similar in each population. Markers heterozygous in both parents (hk × hk) were more represented in population B (41%) than in population A (15%).

**Table 2:** Segregation type by mapping population

	Segregation type in population B				Total population A
	<hk × hk>	<lm × ll>	<nn × np>	Absent	
Segregation type in population A					
< hk × hk>	92	57		683	832 (15%)
< lm × ll>	188	194		1814	2196 (41%)
< nn × np>			80	2265	2345 (44%)
Absent	162	93	209		
Total population B	442 (41%)	344 (32%)	289 (27%)		

Segregation types are in JoinMap format. In brackets, percentage of the total number of SNP by population

Four parental maps and an integrated female map were built. Because of the strong linkages revealed by the pairwise recombination frequencies and LOD scores (Online Resource 2), linkage groups were confidently defined for each parent. The number of linkage groups by parental map ranged from 21 for 74F_B to 26 for Kabusa. This was higher than the *D. alata* base chromosome number (i.e., 20) and may have been the result of the separation of linkage groups containing only a few markers. Nevertheless, the integrated female map (74F) contained 20 linkage groups built using information on both population datasets for each integrated linkage group.

Maps of population B parents were smaller and less dense than those of population A parents. Parental map lengths ranged from 1227 cM for 14 M to 2348 cM for 74F_A, respectively. The map density ranged from one SNP every 2.1 cM in the Kabusa map to one SNP every 3.7 cM in the 74F_B map (Table 3).

**Table 3:** Summary of parental maps

Parent	Map	No. of LGs^1^	No. of SNPs	Length (cM)	Average marker interval (cM)
Female	74F_A	25 (21)	1035	2348	2.30
	74F_B	21 (20)	486	1705	3.70
	74F	20	983	2120	2.20
Male	Kabusa	26 (21)	1078	2195	2.14
	14M	21 (17)	371	1227	3.50

In brackets, the number of linkage groups integrated into the final consensus map

### Map integration and consensus map construction

Male linkage groups were combined with the integrated female map based on the recombination frequencies (Online Resources 3–4). Linkage groups were numbered in reference to the *D. rotundata* genome, as for the integrated female map (74F). Map integration was fairly accurate, as revealed by the good collinearity between homolog linkage groups from the different parental maps (Online Resource 5).

**Fig. 1:**
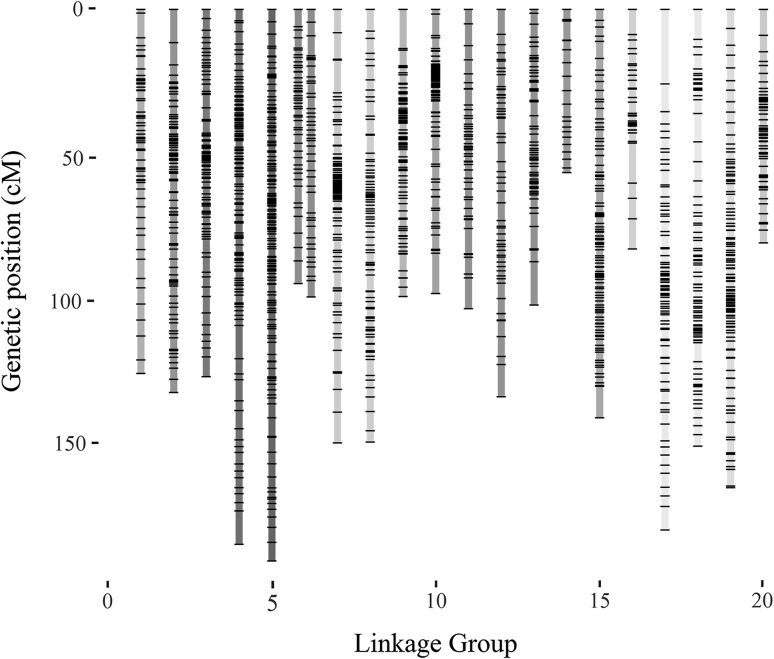
Greater yam (*D. alata* L.) consensus genetic map containing 1548 SNPs. X-axis, linkage groups numbered from LG1 to LG20 homology with the *D. rotundata* reference genome (Tamiru et al. 2017); y-axis, genetic distance (Kosambi mapping function; cM)

The consensus genetic map obtained in this study spanned 2613.5 cM and contained 1579 SNPs distributed on 20 linkage groups, as expected (Fig. 1, Table 4). Linkage groups contained from 20 (LG14) to 145 (LG05) SNPs, with a genetic length ranging from 55.6 cM (LG14) to 188.1 cM (LG05). The mean marker density was one SNP every 1.68 cM. Each SNP had a single position (Table 4).

**Table 4:** Description of the consensus genetic map for *D. alata* L. by linkage groups

Linkage group	No. of SNPs	Genetic length (cM)	Marker interval (cM)^a^	Max gap (cM)	No. of gaps > 5 cM	Physical length (Mb)^b^	Marker interval (kb)^c^
LG01	56	124.2	2.26	8.5	6	28.0	142.3
LG02	88	130.6	1.50	11.3	2	33.9	141.7
LG03	81	125.3	1.57	6.7	3	18.6	120.0
LG04	125	182.5	1.47	11.4	5	29.9	126.7
LG05	145	188.1	1.31	6.4	4	28.5	99.8
LG06_M	47	93.5	2.03	7.7	4	20.8	370.3
LG06_F	42	98.1	2.39	7.0	3	19.4	173.2
LG07	88	147.8	1.70	11.2	8	19.3	123.0
LG08	80	147.6	1.87	7.4	8	20.3	117.0
LG09	90	98.0	1.10	13.3	3	27.6	133.3
LG10	72	96.9	1.36	13.7	3	18.1	88.6
LG11	47	102.1	2.22	10.5	5	15.7	209.1
LG12	56	132.1	2.40	11.0	9	28.9	73.5
LG13	65	100.9	1.58	14.8	2	24.6	220.6
LG14	20	55.6	2.93	6.6	1	11.7	139.7
LG15	102	139.3	1.38	10.8	2	18.5	99.6
LG16	31	81.7	2.72	13.7	5	2.8	26.4
LG17	83	177.5	2.17	25.4	4	21.3	165.1
LG18	88	149.0	1.71	10.2	6	29.4	133.2
LG19	117	163.2	1.41	6.5	4	39.7	132.4
LG20	56	79.6	1.45	8.8	2	13.0	89.1
Total	1579	2613.5	1.68			442.1	139.1

For the sex-related LG6, two maps were conserved: the female-integrated map (LG6_F) and a male consensus map (LG6_M)

^a^Mean distance between SNPs

^b^Total length after reordering the *D. rotundata* reference genome (Tamiru et al. 2017) according to *D. alata* consensus map

^c^Median physical distance between SNPs in the reordered *D. rotundata* genomic sequence

One linkage group of the 74F_A map that could not be included in the integrated female map but had sufficient common SNPs with male maps was also integrated in the final consensus map (Online Resource 3). The consensus map did not contain information of male B (14 M) for three linkage groups (LG3, LG4 and LG16) as no homologs were found (Online Resource 4). The male A (Kabusa) homolog linkage group of LG14 was constructed but did not contain sufficient bridge markers with the other three parental homologs of LG14 to be integrated into the final consensus map (Online Resource 4).

For LG6, two maps were included in the final consensus map: the integrated female map of LG6 (LG6_F) and a male consensus map of LG6 (LG6_M). Indeed, map integration of the different LG6 homologs was not possible because the male maps did not contain any common SNPs with the female consensus map. As dioecy can be related to the chromosome architecture, the relation between these map integration issues and sex determination was further investigated.

### Detection of sex-determining QTLs

The phenotypic data analysis revealed no significant differences between the observed sex ratio and a 1:1 theoretical ratio within both populations (χ^2^ test; population A, *P* value = 0.55; population B, *P* value = 0.13), indicating that sex determination may be controlled by one dominant allele.

Combining genotypic and phenotypic datasets, QTL detection was performed on fewer progenies and conducted on parental maps with 42 (19 females + 23 males) and 60 (27 females and 33 males) progenies for populations A and B, respectively. Only one QTL per population was detected on LG6 homologs and only in the male maps (Online Resource 6A). No QTLs were detected using the female maps. In agreement with the sex ratio observed within both populations, these findings suggest that only one locus may be involved in sex determination and may be inherited via a system of XX/XY sex chromosomes involving heterogametic males (XY).

**Fig. 2:**
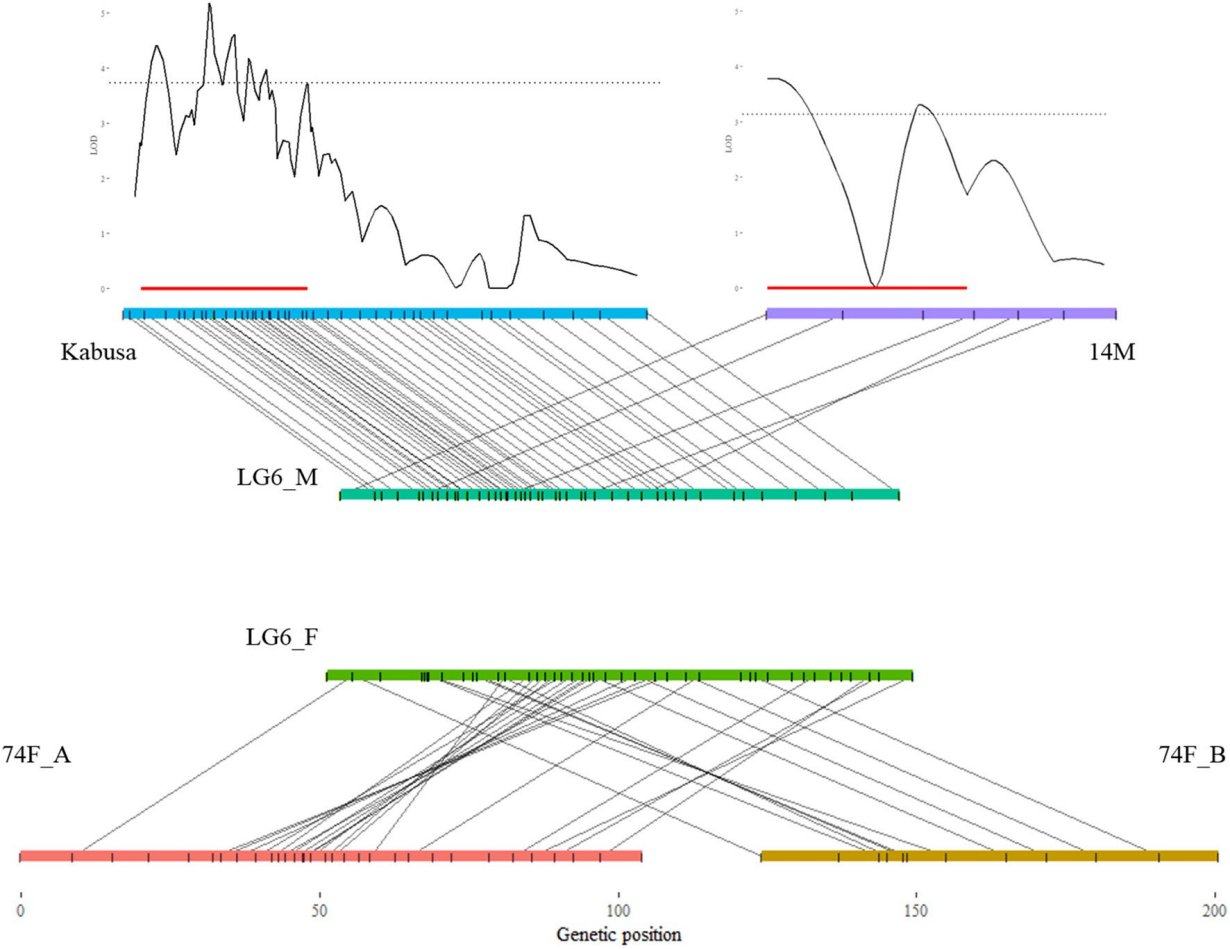
Homology between parental genetic maps of LG6 homologs and detection of the sex-determining QTLs. On LOD score plots: dashed lines, LOD score threshold; red lines, QTL confidence interval. On linkage groups: black ticks, SNPs position

For male A (Kabusa), the QTL confidence interval spanned from 1.1 to 30.2 cM, with a peak LOD score of 5.2 located at 13.0 cM. The nearest SNP (06.1_27885348) located at 13.3 cM allowed us to predict sex in 85% of the cases (Fig. 2). For male B (14 M), the QTL confidence interval spanned from 0 to 34.8 cM, with a peak LOD score of 3.8 located at 1.0 cM. The LOD drop in the middle of the QTL interval was due to a marker phase change. The nearest SNP (06.1_27950405) located at 0.0 cM allowed us to predict sex in 77% of the cases (Fig. 2). In both maps, the QTL peaks contained tightly linked SNPs (Online Resource 6B). In both cases, no secondary peaks were found when the most significant SNPs were used as covariates. Once projected on the consensus male map (LG6_M), the two QTL intervals co-localized and were located between 4.8 and 33.9 cM and between 0 and 34.8 cM for male A and male B, respectively. The locus involved in sex determination in the two populations may be the same.

### *D. alata* scaffold anchoring and synteny with *D. rotundata*

Based on the SNP positions in our consensus *D. alata* genetic map compared to SNP positions in the *D. rotundata* genome, the *D. rotundata* genome was reordered: (i) to highlight possible chromosome rearrangements between the two species and (ii) to estimate the relationship between physical and linkage map distances in *D. alata*.

**Fig. 3:**
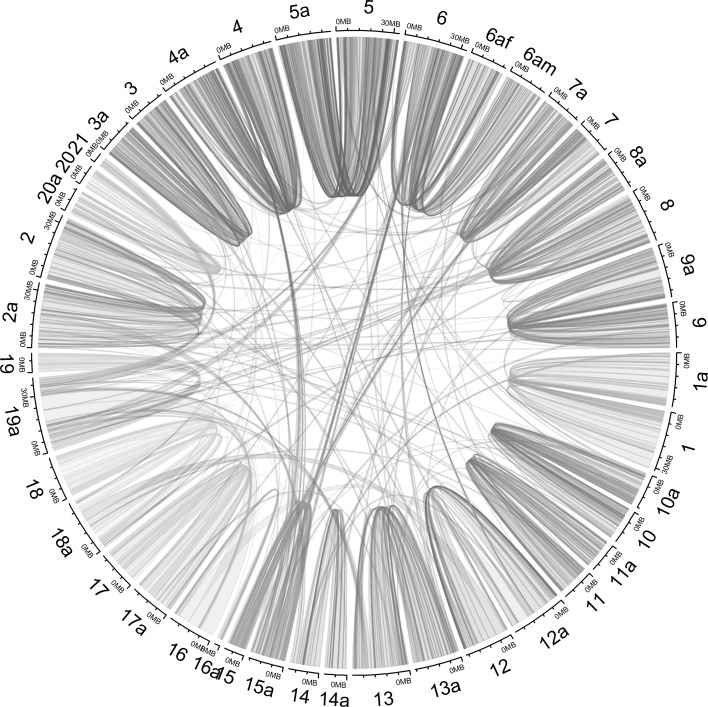
Synteny between *D. alata* and *D. rotundata*. Chromosomes numbered with the suffix ‘a’ are for *D. alata* corresponding to the *D. rotundata* reference sequence reordered according to our final consensus map. For LG6, both female (6af) and male (6am) reordered chromosomes were conserved

The mean homology between *D. alata* and *D. rotundata* linkage groups was 87% when computed as the percentage of SNPs located in a *D. alata* linkage group and in its *D. rotundata* homolog (Online Resource 7). The highest homology between the two yam species was found for LG5 and LG8, with 99% of SNPs from *D. alata* belonging to the same respective linkage group in *D. rotundata* (Fig. 3; Online Resource 7).

*D. alata* LG19 was the less conserved, with only 48% of SNPs belonging to *D. rotundata* chromosome 19 (Online Resource 7). This could partially be explained by the fact that during *D. rotundata* sequencing a supernumerary chromosome 21 was assumed which was mapped at the end of our LG19 (Fig. 2). In our study, LG19 also included 10% of SNPs located on chromosome 16 of *D. rotundata* (Online Resource 6) while spreading over 63% of this chromosome (Online Resource 8). In our results, LG16 contained few SNPs (31) and was the smallest, with a total estimated length of 2.8 Mb (Table 4; Fig. 3).

LG15 and LG7 also appeared to be rearranged regarding the percentage of SNPs (Online Resource 7). This was even truer for LG15 when homology was estimated on the basis of the percentage of genomic sequence mapped on its homolog chromosome (Online Resource 8). Indeed, the reordered chromosome corresponding to LG15 was composed of *D. rotundata* chromosomes 15 (43%), 6 (26%) and 4 (11%) (Fig. 3).

The median SNP interval was 139.1 kb in our reordered *D. rodundata* genome (Table 4). SNP intervals ranged from 26.4 kb for LG16 to 370.3 kb for LG6_M. For most linkage groups, the relationship between the physical and the linkage map distances varied along linkage groups, in agreement with an expected chromosomal structure made of highly recombinant telomeres and a less recombinant centromere (Online Resource 9).

*D. alata* has not yet been completely sequenced. However, useful genomic resources have been released upon which our SNPs could be positioned. Overall, 743 scaffolds from the *D. alata* genome v1 were anchored in the consensus maps, representing around 40% (115.8 Mb/287.3 Mb) of the total scaffold length (Online Resource 10). Around 38.5% (286/743) of these scaffolds could theoretically be oriented as they contained two or more SNPs. The scaffolding and linkage analysis findings seemed quite consistent, as revealed by the fact that 74.5% (213/286) of the scaffolds containing two or more SNPs contained only SNPs mapped on the same LG.

## Discussion

### A *D. alata* reference map

The method used to build the reference genetic map was designed to be conservative. Indeed, integration of maps into the final consensus map was based on pairwise recombination frequencies in parental datasets and not on map projections. All SNPs mapped in parental maps were thus not necessarily included in the final reference map and vice versa. Although this procedure is more restrictive, it was applied to minimize the impact of errors due to the reversal of locations for short distance markers and/or of structural variations (Mace et al. 2009; Khan et al. 2012). In the same conservative spirit, highly distorted markers were eliminated before linkage analysis (Zhang et al. 2010).

Our consensus map is the first SNP high-density genetic map for *D. alata*. It contains 20 linkage groups, as expected, and 1579 SNPs spread over 2613.5 cM, with an average marker interval of 1.7 cM. Indeed, using the same Kosambi mapping function, the previously published map lengths were 1233 cM, 1538 cM and 3229.5 cM, and they contained 494, 523 and 380 markers with an average marker spacing of 2.6 cM, 3.3 cM and 14.2 cM according to Mignouna et al. (2002), Petro et al. (2011) and Bhattacharjee et al. (2018), respectively. The genome coverage was estimated at 65% by Mignouna et al. (2002) and 80% by Petro et al. (2011). The estimated genome coverage of our map reached 94% using Method 4 of Chakravarti et al. (1991).

Diploid *D. alata* genotypes have a haploid genome size estimated to be between 1C = 0.46 pg = 454 Mb (Arnau et al. 2009) and 1C = 0.57 pg = 562 Mb (Obidiegwu et al. 2010) by flow cytometry analysis. Saski et al. (2015) de novo sequencing resulted in an assembly of contigs covering 428.9 Mb. These estimations are slightly smaller than the *D. rotundata* genome size (570–579 Mb; Tamiru et al. 2017). Moreover, physical distance in *D. rotundata* and *D. alata* seems well correlated and proportional to smaller distance for *D. alata* (*r*^2^ = 0.79; coefficient of proportionality = 0.786; Online Resource 11). The good resolution (in kb) of our map thus may have been fairly well estimated or even slightly underestimated in this study.

In this sense, our estimated marker density of one SNP every 139.1 kb agreed with the fact that around 40% of the total assembly of the *D. alata* genome v1 was contained in our reference map. Indeed, presently 50% of this pre-release assembly is composed of scaffolds longer than 145.7 kb (Water Yam Genome Project—ftp://yambase.org/genomes/Dioscorea_alata/). In *D. rotundata*, 89.6% of the genome assembly is included in scaffolds longer than 200 kb (Table S3 in Tamiru et al. 2017). If the ongoing *D. alata* sequencing results in similar scaffolding, our reference map may be sufficient to order most of the future assembly.

More generally, this genetic map for greater yam, associated with the reordered *D. rotundata* genome and the mapping of available *D. alata* scaffolds, will promote further investigations on the inheritance of key traits and the development of molecular breeding tools. It will also help gain further insight into yam evolution and facilitate the transfer of knowledge regarding different yam species. We thus strongly encourage retaining the linkage group nomenclature we used for *D. alata* as it is also based on *D. rotundata* nomenclature.

#### Segregation distortion

The difference in linkage analysis power between population A and population B mostly resulted from the lower number of SNPs available for mapping in population B. Three consensus linkage groups of the final consensus map did not contain information on the population B male parent (14 M) as no homologs were found. Moreover, parental maps derived from population B were shorter and less dense than those derived from population A.

First, for a similar GBS quality (i.e., number of produced reads and missing data), fewer SNPs were detected in population B than in population A. This agreed with the genetic proximity between 74F and 14 M (76% of shared alleles) compared to that of 74F and Kabusa (65% of shared alleles). Then, the segregation distortion threshold excluded a higher proportion of markers in population B than in population A. Segregation distortions have been widely reported in plant species including *D. alata*. In Petro et al. (2011), 19% of markers were tagged as distorted, while in Bhattacharjee et al. (2018) it was 39.8%. However, comparisons are limited due to the unknown threshold used to test distortion.

Regarding the decrease in the number of progenies from hybridization to mapping datasets, one major hypothesis could be proposed. Indeed, the main differences were the ratio of introduced embryos to the number of pollination between population A (140/250 = 56%) and population B (280/360 = 78%), which was related to gametophyte selection, and the proportion of rescued embryos successfully brought to the field (population A, 121/140 = 86%; population B, 193/280 = 69%), which was related to early-stage zygotic selection. However, the sampling bias—estimated when considering the proportion of non-genotyped progenies and introduced by filters on data quality per progeny—was similar between the two populations. Thus, we hypothesized that early-stage zygotic selection affected to a greater extent population B for which segregation distortion was mainly related to genes involved in the response to in vitro and field stresses. Regarding the smaller size of population A compared to population B, segregation distortion may have been more related to the sampling bias within population A. This hypothesis agrees with the relative genetic proximity of 74F and 14 M, and the inbreeding depression observed for seed germination and zygotic viability in *D. alata* breeding programs (Abraham et al. 2006).

#### *Sex determination in* Dioscorea

Dioecy is a key character in *Dioscorea* species (Fig. 2 in Viruel et al. 2016). Based on cytological observations, previous studies have mostly reported an XX/XY chromosome system (review in Martin 1966), indicating that Y is the sex-determining chromosome and males are heterogametic. When assessing the sex ratio in a test-cross design, an XX/XY chromosome system was also proposed for *D. floribunda* (Martin 1966) and for the dioecious *D. tokoro* using QTL detection with AFLP genetic maps (Terauchi and Kahl 1999). Our results agreed with a XX/XY sex-determining system in *D. alata* mapped at the same location (beginning of LG6_M) in the two male parents. This conclusion has to be confirmed using a more diverse range of genotypes to ensure that the XX/XY system we discovered was not specific to the female parent (74F).

The main exception to the XX/XY system was found for the trioecious (mostly dioecious) *D. rotundata* species (Tamiru et al. 2017) for which a ZW/ZZ system (heterogametic female) mapped at the beginning of pseudo-chromosome 11 was described. Indeed, the authors conducted bulk segregant analysis in a biparental population and identified SNPs linked to sex heterozygous in the female parent but not in the male parent (see Fig. 4C in Tamiru et al. 2017). They also identified female-specific regions for which they developed a PCR primer pair. Interestingly, the beginning of pseudo-chromosome 11, which is linked to sex determination in *D. rotundata*, seemed to be rearranged in *D. alata* as it mapped to LG11, but also to LG2, LG18, LG19 and LG6_M (Online Resource 10). The change from cosexuality to dioecy implies a complex evolutionary process consisting of successive mutations for male and female sterility and sex chromosome rearrangement (Charlesworth 2002, 2015; Otto et al. 2011; Kumar et al. 2014). Moreover, the mostly dioecious species *D. rotundata* belongs to the same section (Enantiophyllum) as the strictly dioecious *D. alata*, and there is good synteny and sequence homology between the two species. We thus suggest that their sex determination systems may be related (e.g., transition from XY into ZW system; Kumar et al. 2014).

So far, heteromorphic sex chromosomes have been identified in around half of the species for which sex chromosomes were detected (Hobza et al. 2017). Heteromorphic sex chromosomes have been reported in *Dioscorea* (Martin 1966), but there is still no cytological evidence due to the small size of *Dioscorea* chromosomes. Our results revealed a sex-linked QTL interval larger than 30 cM on the Y chromosome (LG6_M) spreading over approximately 10 Mb. The size of the confidence interval could be related to the small population size used for QTL detection due to the erratic flowering pattern of *D. alata* (Malapa et al. 2005). However, it could also be related to a sex-linked region with a low recombination rate. Indeed, the establishment of sex-determining regions associated with local suppression of recombination in Y chromosomes is the key driver of chromosome Y differentiation (Otto et al. 2011; Kumar et al. 2014; Hobza et al. 2015). In this sense, LG6_M (Y) is the linkage group with the lowest resolution (kb^−1^) and contains no common marker with LG6_F (X). In *D. tokoro*, Terauchi and Kahl (1999) showed that all markers of the Y chromosome spreading over 23.3 cM showed tight linkage to sex compared to the absence of sex-linked markers on the X chromosome spreading over 82.5 cM.

## Conclusion

Linkage analysis studies on two biparental populations were combined to build a high-density SNP genetic map of greater yam (*D. alata*). This map covered 94% of the genome and contained 1579 SNPs. Regarding sequence homology and synteny with its already sequenced relative *D. rotundata*, a reordered *D. rotundata* genome adapted to *D. alata* was proposed. The goal was: (i) to facilitate further investigations on the identification of loci linked to key traits, history and evolution and (ii) to enhance knowledge transfer within the *Dioscorea* genus. Indeed, the estimated resolution of this map was 139.1 kb, thus allowing QTL and gene cloning strategies. Based on our study, we also encourage a common linkage group nomenclature. Information on female and male LG6 carrying a major locus determining sex was separately conserved within this consensus map. Indeed, sex ratio analysis within populations and QTL detection revealed a XX/XY sex chromosome system, and the presence of heteromorphic sex chromosomes could reasonably be hypothesized.

### Author contribution statement

GA designed the study with the support of HC. EM, EN, GA and MCG created and maintained the plant material. FC, HC, HV and PM generated the genotyping-by-sequencing data and their analysis. EN, EM and FC phenotyped the progenies. FC and GA conducted linkage analysis. FL and FC performed the QTL mapping. FC, GA, HC, PM and FL wrote the manuscript.

## Electronic supplementary material

Below is the link to the electronic supplementary material.

Supplementary material 1 (PDF 3121 kb)

Supplementary material 2 (XLSX 431 kb)
