# Supplementary material for: A reference high-density genetic map of greater yam (Dioscorea alata L.)
Source: Theor Appl Genet. 2019 Feb 20;132(6):1733–44. doi: 10.1007/s00122-019-03311-6 (PMC6531416; doi:10.1007/s00122-019-03311-6)

Online resource 1: Summary of sequencing results (GBS) and mapping quality. Sequencing reads were mapped to the *D. rotundata* reference genome (pseudo chromosomes BDMI0100001-21; Tamiru *et al*., 2017). SNP discovery was conducted using the VcfHunter package (Garsmeur *et al*., 2018; available at https://github.com/SouthGreenPlatform/VcfHunter/).

	**74F**	**Kabusa**	**14M**	**PopA**	**PopB**
**Total # of reads**	25,528,679	19,161,000	21,223,418	530,432,716	720,572,466
**Mapping on Genome reference: aligned 0 times**	3,930,774	3,399,813	3,254,317	80,339,970	112,524,234
**Mapping on Genome reference: aligned exactly one time**	19,331,058	14,005,832	15,951,780	401,338,915	546,418,237
**Mapping on Genome reference: aligned > one time**	2,266,847	1,755,355	2,017,321	48,753,831	61,629,995
**Overall alignment rate**	84.9%	82.2%	84.2%	83.4%	82.3%

Online resource 2: Recombination frequencies and LODs by parental maps. Upper part, recombination frequencies; lower part, LOD. SNPs are ordered thanks to their position in the parental maps. Figures resulted from the R/qtl 1.42-8package (Broman *et al*., 2013).

Female A – 74F_A

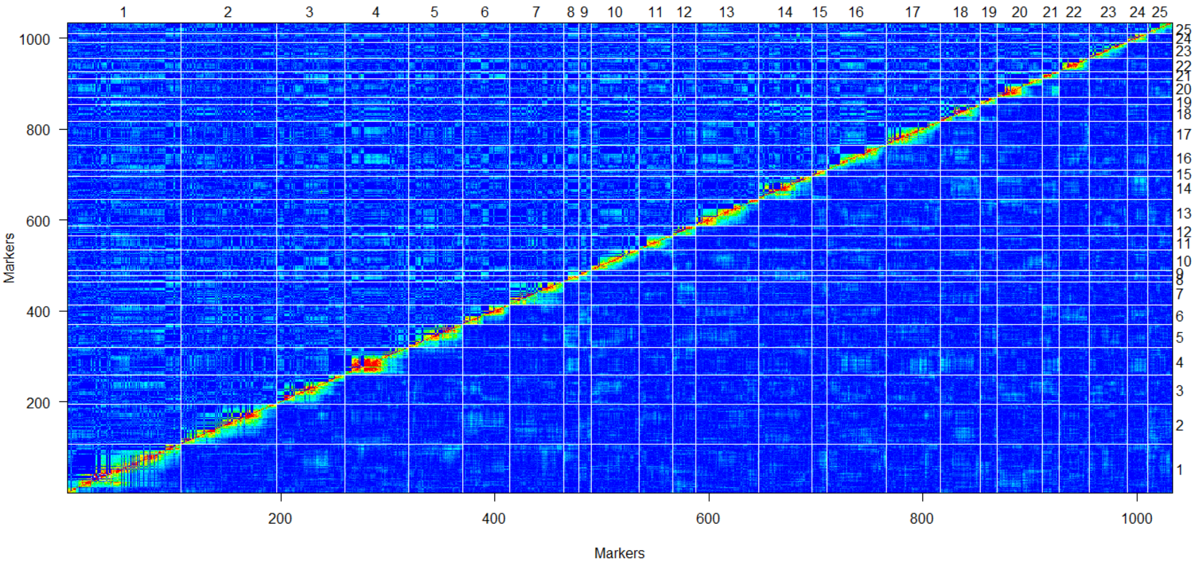

Female B – 74F_B

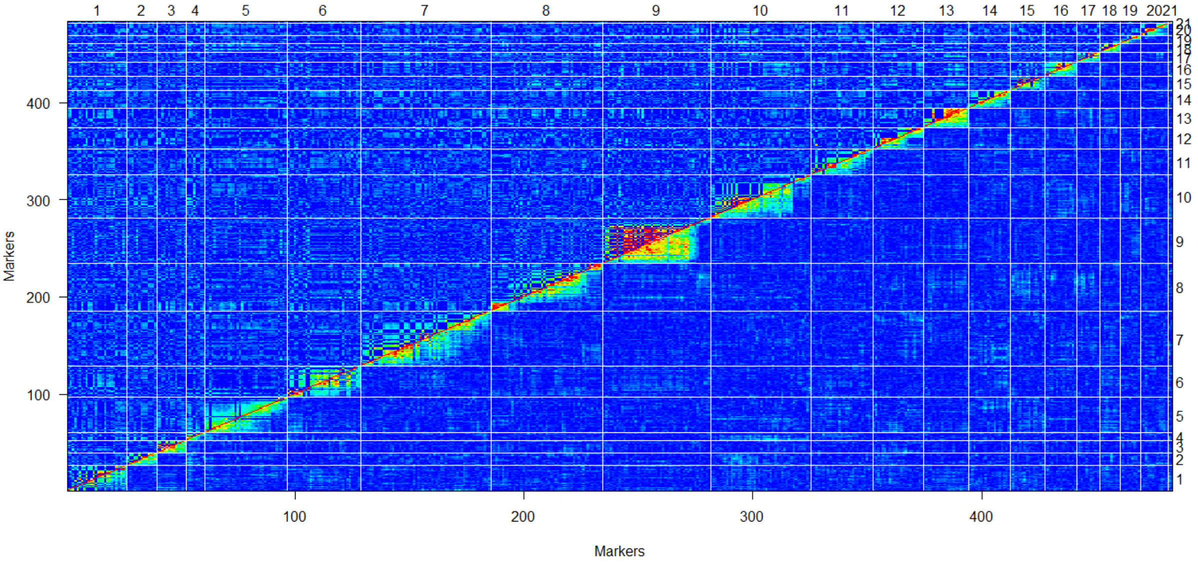

Male A - Kabusa

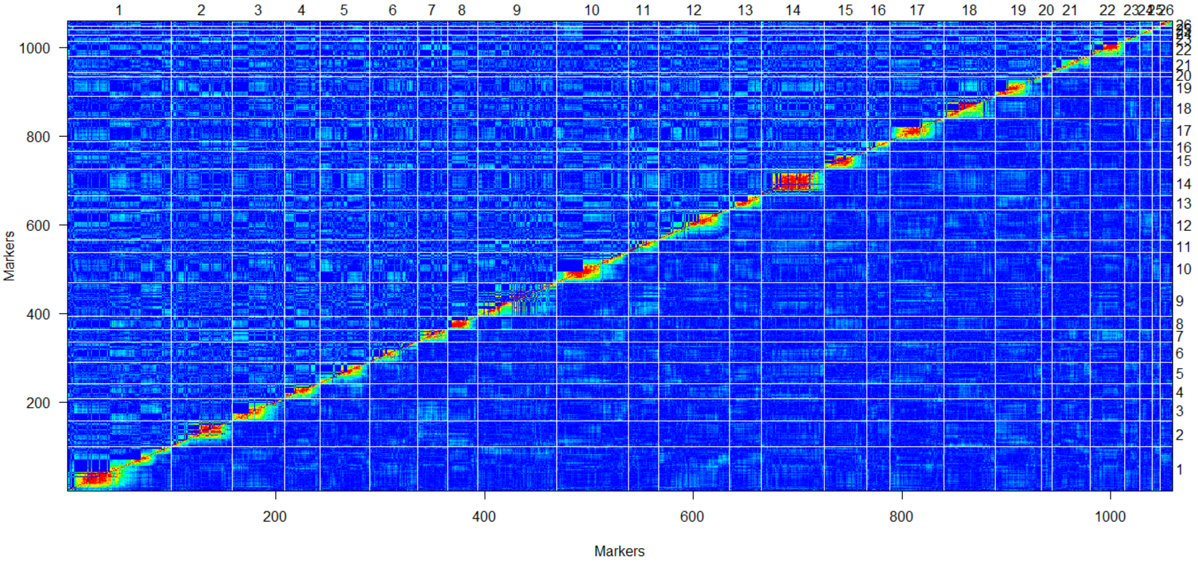

Male B – 14M

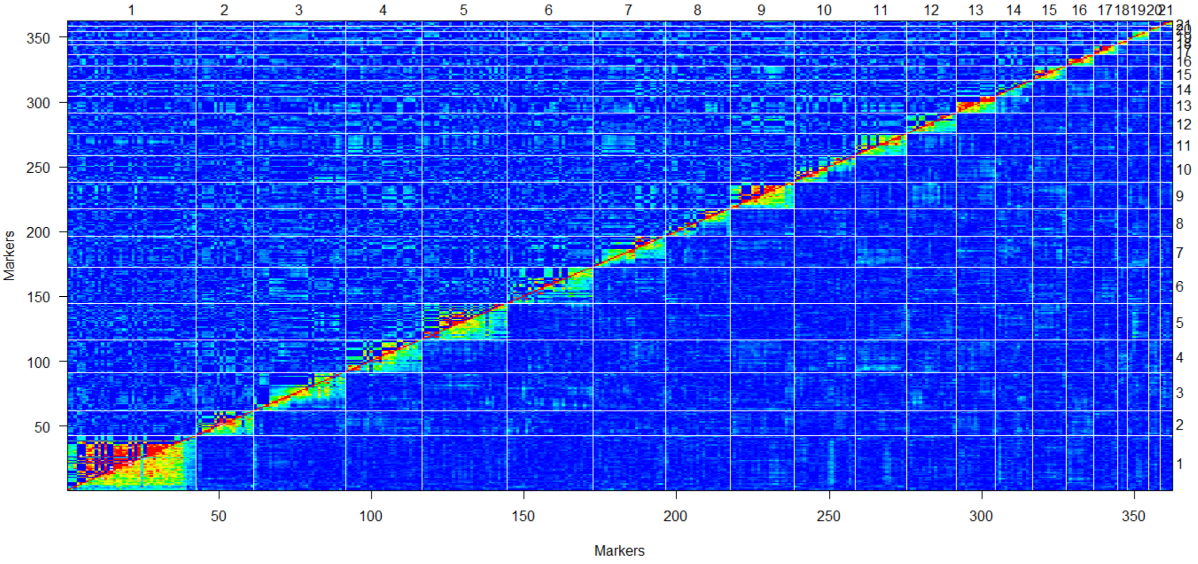

Online resource 3: Number of common SNPs between groupings defined in the linkage analysis.

		Female B (74F_B) grouping																				
		18	9	5	14	7	20	12	2	6	21	13	16	11	17	10	19	15	1	8	3	4
Female A (74F_A) grouping	23	7																				
	17		34																			
	13			24																		
	14				11																	
	1					45																
	11						9															
	16							17														
	10								10													4
	4									26												
	25										2											
	7											18										
	6												8									
	5													21								
	8														7							
	3												1			25						
	24																4					
	20																	11				
	18																		23			
	2																			39		
	22																				8	
	12																					
	15																					
	19																		1			
	21																					
	9														2							

		Male A (Kabusa) Grouping																									
		11	6	7	1	2	23	19	14	17	18	8	13	20	21	10	12	16	5	9	3	4	22	15	24	25	26
Integrated female (74F) grouping	1	22																									
	2		52																								
	3			15																							
	4				21																						
	5					112	9																				
	6							4																			
	7								40																		
	8									21																	
	9										60																
	10											1															
	11												14														
	12													5													
	13														24												
	14																										
	15																43										
	16																	13						1			
	17																		10								
	18																			36							
	19																				27	18					
	20																						15				

		Male B (14) grouping																				
		11	1	8	19	17	3	10	18	9	15	6	12	5	7	4	16	13	14	2	20	21
Integrated female (74F) grouping	1	15																				
	2		60																			
	3																					
	4																					
	5			24																		
	6																					
	7					11																
	8						5															
	9							29														
	10								6													
	11									29												
	12										14											
	13											21										
	14												12									
	15													30								
	16																					
	17														21							
	18															26						
	19																13	16	16			3
	20																			15		

Online resource 4: Summary of linkage group integration during consensus map creation. Linkage groups of 74F_A and 74F_B maps were integrated in the 74F map. Then, 74F was integrated with Kabusa, 14M, and Other to create the consensus map. In the integrated female map (74F) and in the consensus map, linkage groups were numbered by homology with the *D. rotundata* reference genome (Tamiru *et al*., 2017). In italic, linkage groups for which integration into the final consensus map failed due to insufficient linkage (*i.e.* linkage group 10 in Kabusa map).

74F_A	74F_B	**74F**	Kabusa	14M	Other	**Consensus Map**
23	18	**1**	11	11		**1**
17	9	**2**	6	1		**2**
13	5	**3**	7			**3**
14	14	**4**	1			**4**
1	7	**5**	2 + 23	8		**5**
11	20	**6_F**	19	19		**6_M**
16	12	**7**	14	17		**7**
10	2	**8**	17	3		**8**
4	6	**9**	18	10		**9**
25	21	**10**	8	18		**10**
7	13	**11**	13	9		**11**
6	16	**12**	20	15		**12**
5	11	**13**	21	6		**13**
8	17	**14**	*10*	12	9 (74F_A)	**14**
3	10	**15**	12	5		**15**
24	19	**16**	16			**16**
20	15	**17**	5	7		**17**
18	1	**18**	9	4		**18**
2	8	**19**	3 + 4	16		**19**
22	3	**20**	22	2		**20**

Online resource 5: Collinearities between the different parental and consensus maps by homolog linkage groups. Linkage groups are numbered in reference to *D. rotundata* genome sequence (Tamiru *et al*., 2017). For each linkage group, from up to down and right to left, 74F_A (orange, position 1), 74F_B (yellow, position 1), 74F (integrated female map, green, position 2), reference (turquoise, position 2.5), Kabusa (male A, blue, position 5) and 14M (male B, pink, position 5). Common SNPs are connected by black lines.

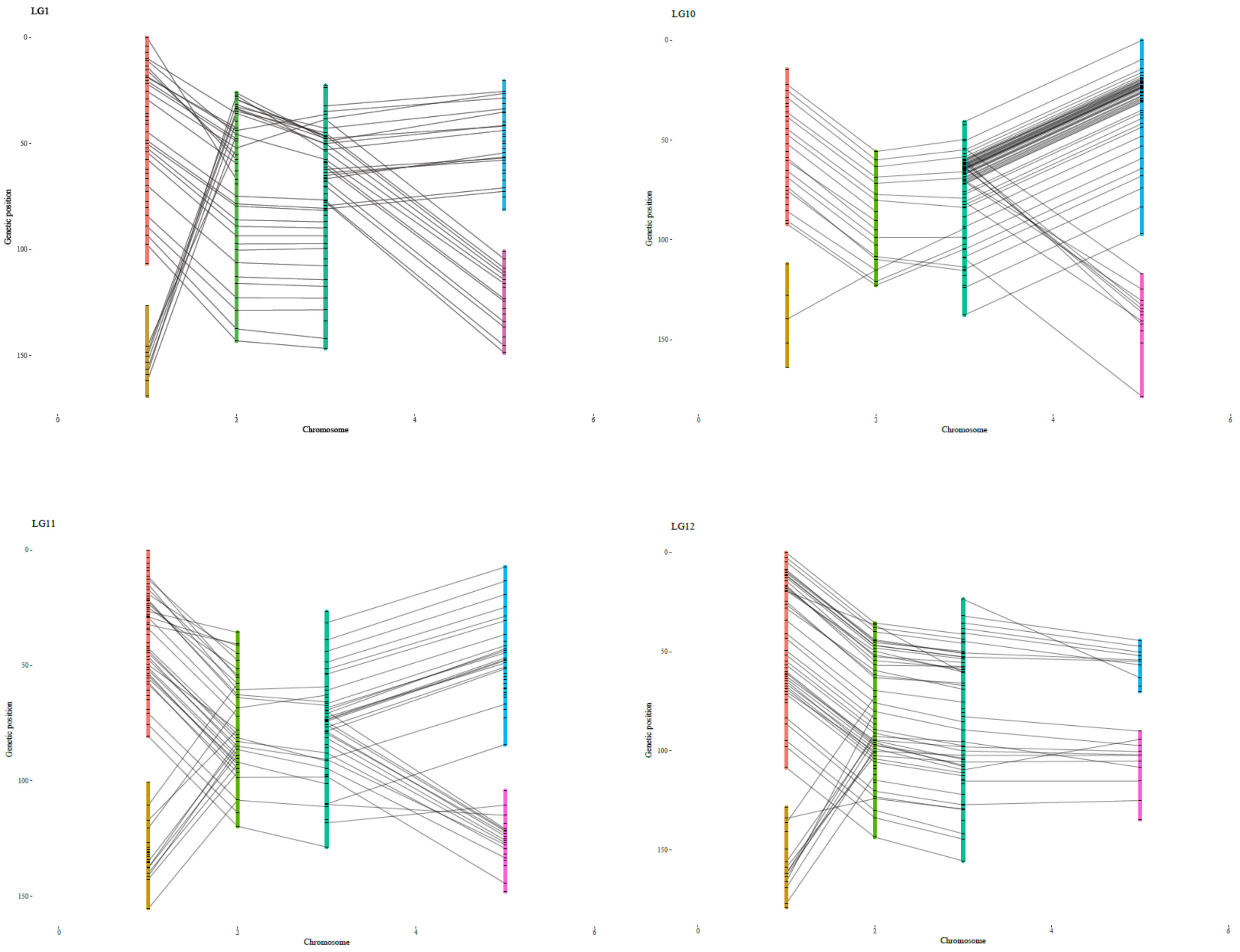

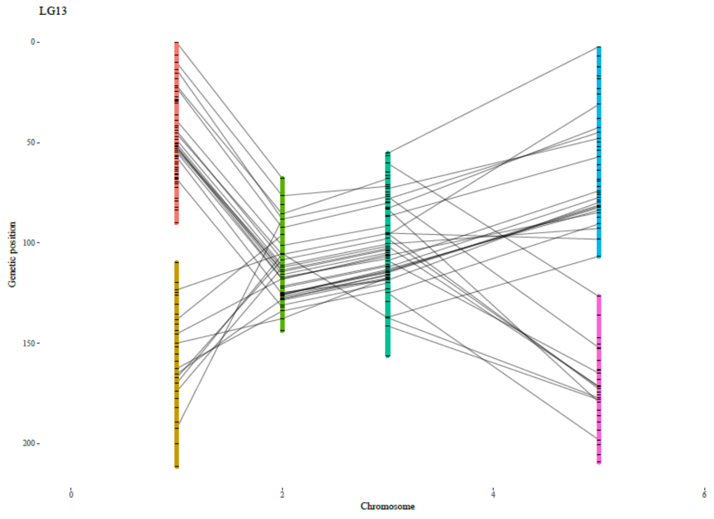
LG13
Genetic position
0
50
100
150
200
Chromosome
0
2
4
6

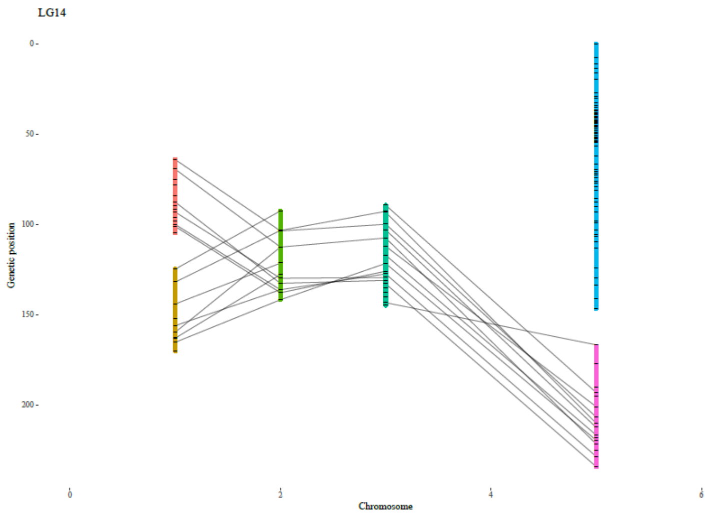
LG14
Genetic position
0
50
100
150
200
Chromosome
0
2
4
6

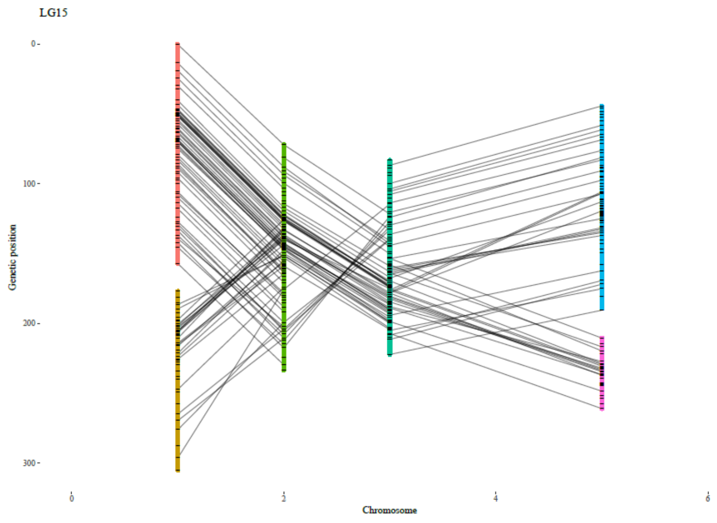
LG15
Genetic position
0
100
200
300
Chromosome
0
2
4
6

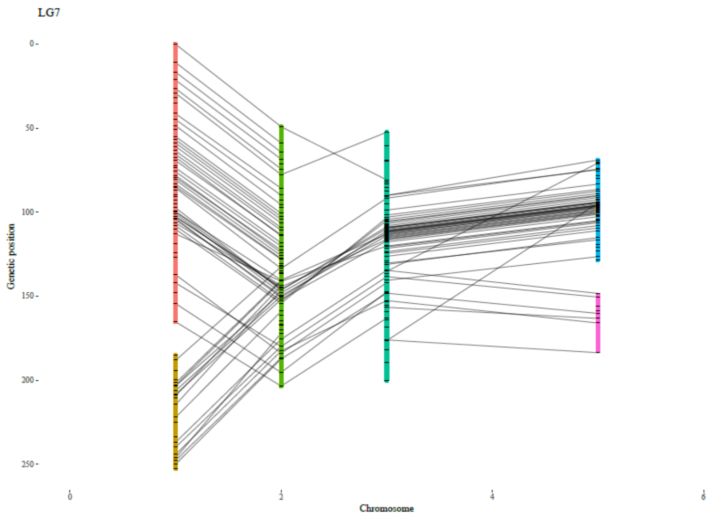
LG7
Genetic position
0
50
100
150
200
250
Chromosome
0
2
4
6

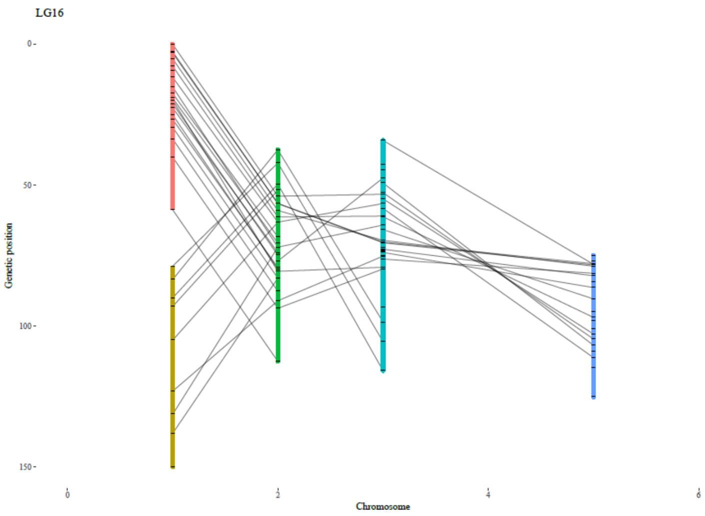
LG16
0
50
100
150
Genetic position
0
2
4
6
Chromosome

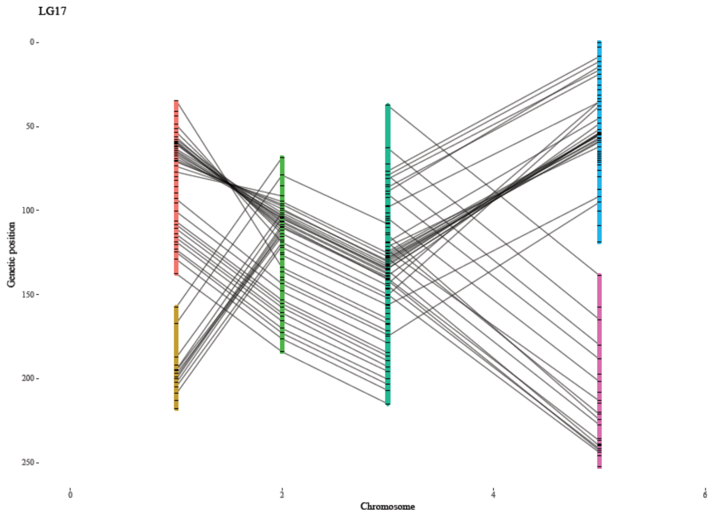
LG17
0
50
100
150
200
250
Genetic position
0
2
4
6
Chromosome

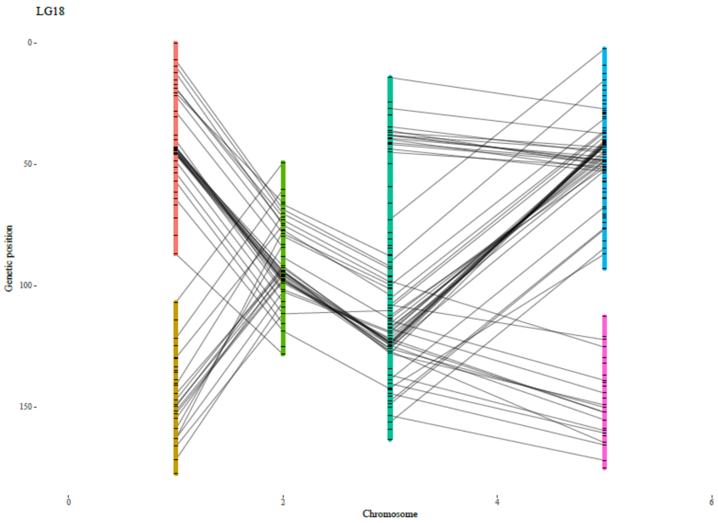
LG18
0
50
100
150
Genetic position
0
2
4
6
Chromosome

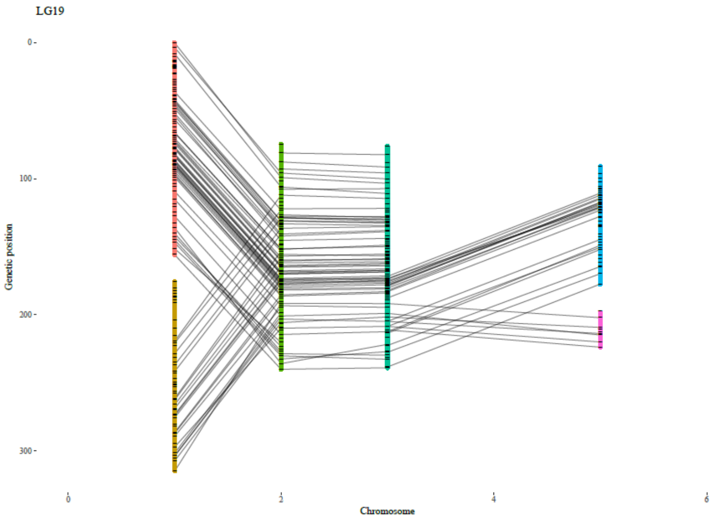
LG19
0
100
200
300
Genetic position
0
2
4
6
Chromosome

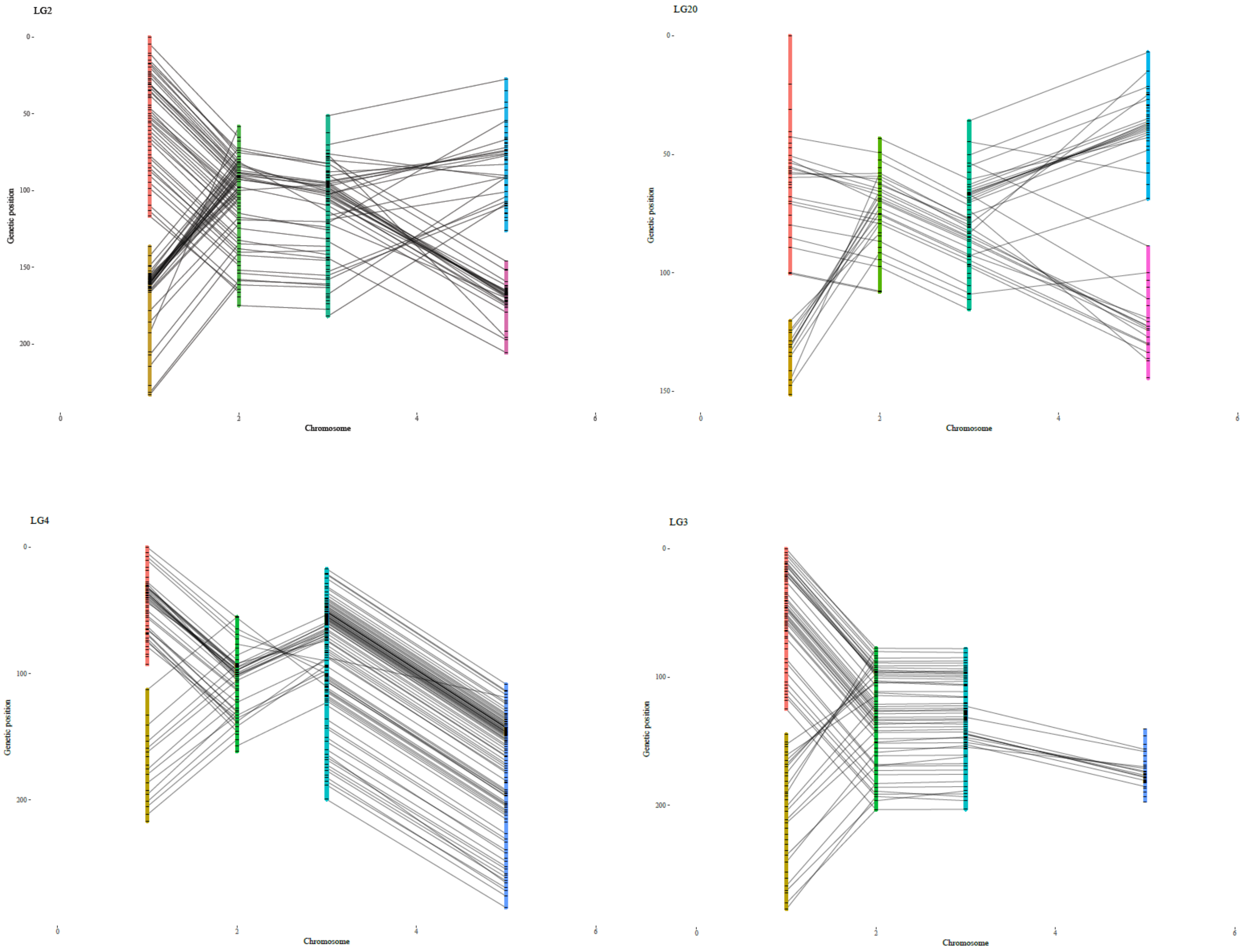
LG2
Genetic position
0
50
100
150
200
Chromosome
0
2
4
6
LG20
Genetic position
0
50
100
150
Chromosome
0
2
4
6
LG4
Genetic position
0
100
200
Chromosome
0
2
4
6
LG3
Genetic position
0
100
200
Chromosome
0
2
4
6

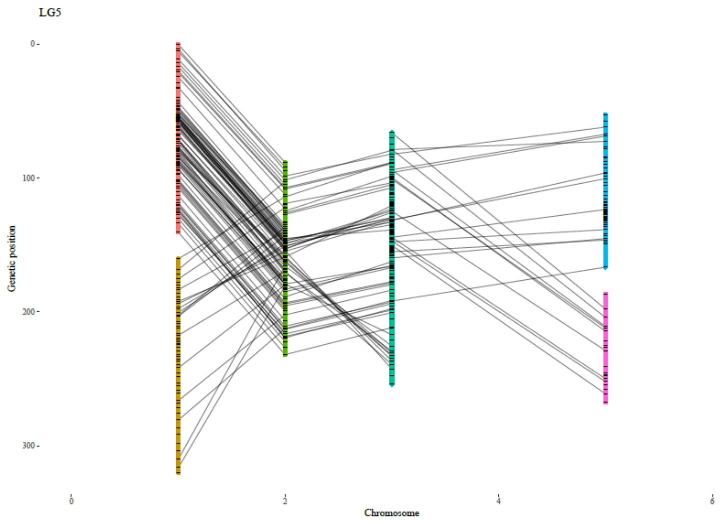
LG5
0
100
200
300
Genetic position
0
2
4
6
Chromosome

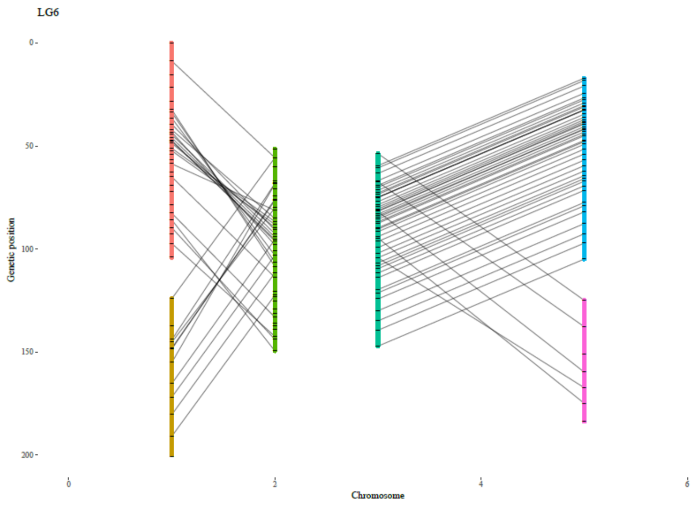
LG6
0
50
100
150
200
Genetic position
0
2
4
6
Chromosome

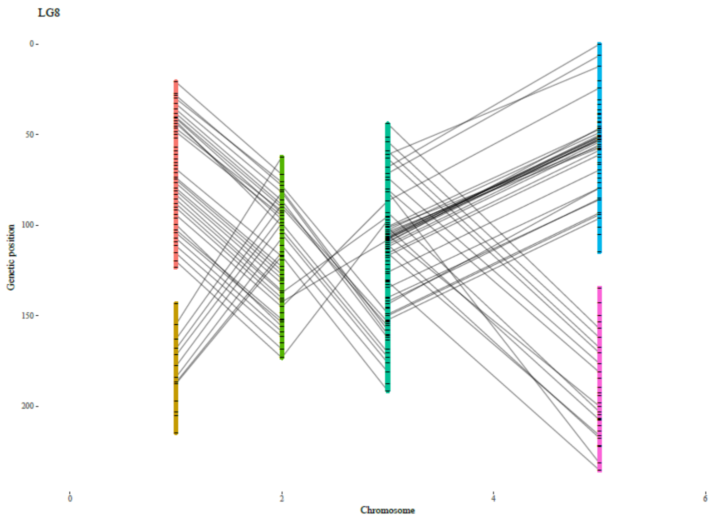
LG8
0
50
100
150
200
Genetic position
0
2
4
6
Chromosome

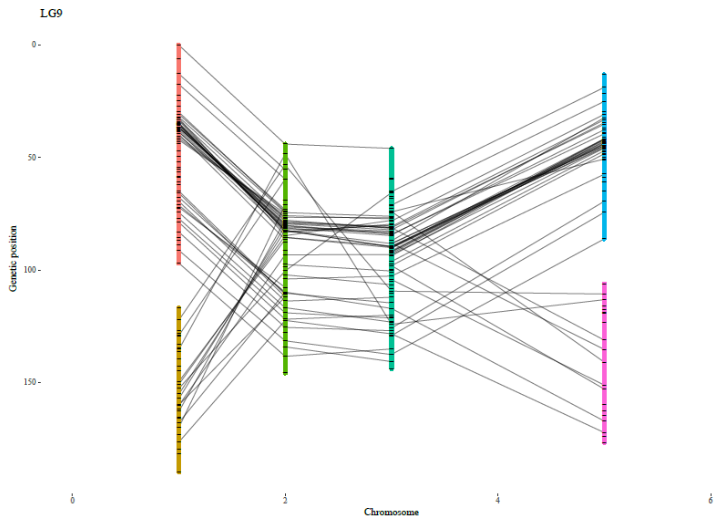
LG9
0
50
100
150
Genetic position
0
2
4
6
Chromosome

Online resource 6: A) details on detection of sex-determining QTLs on parental maps and B) details on significant QTLs detected on male parental maps.

Online resource 6A - LOD score plots by parental LGs. QTL detection was conducted using the R/qtl 1.42-8 package (Broman et al., 2013) and a simple interval mapping approach (option: step = 1 cM, error.prob = 1e-08, map.function = "kosambi", model = "binary", method = "hk"). Significant QTLs were detected only for LG19 in male A (Kabusa) and LG19 in male B (14M) which were the two linkage groups integrated into the LG6_M consensus map. No significant QTL were encountered when detection was conducted on female maps.

Male A - Kabusa

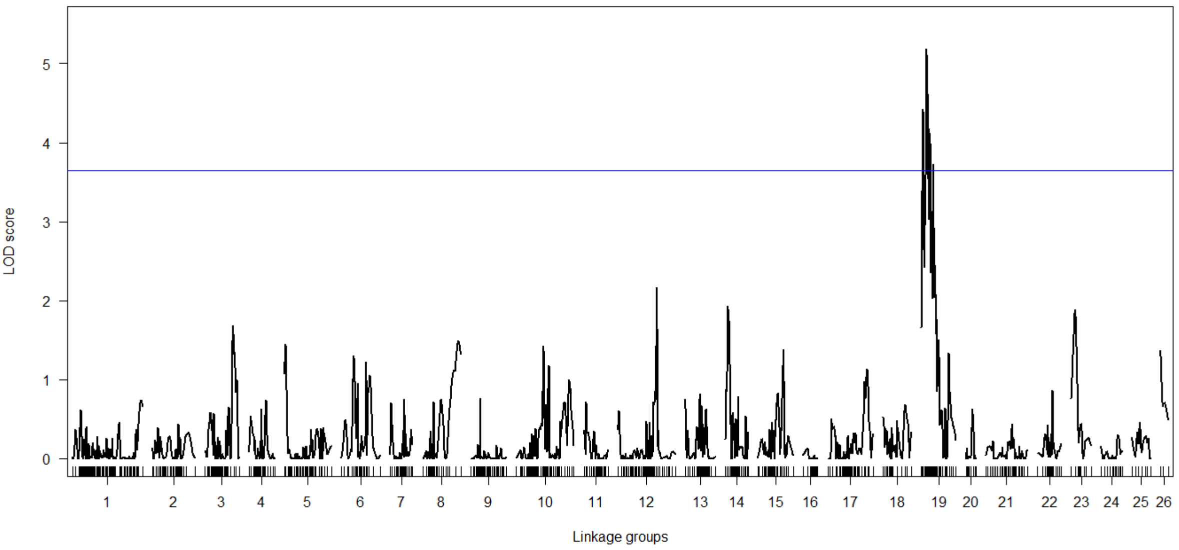

Male B – 14M

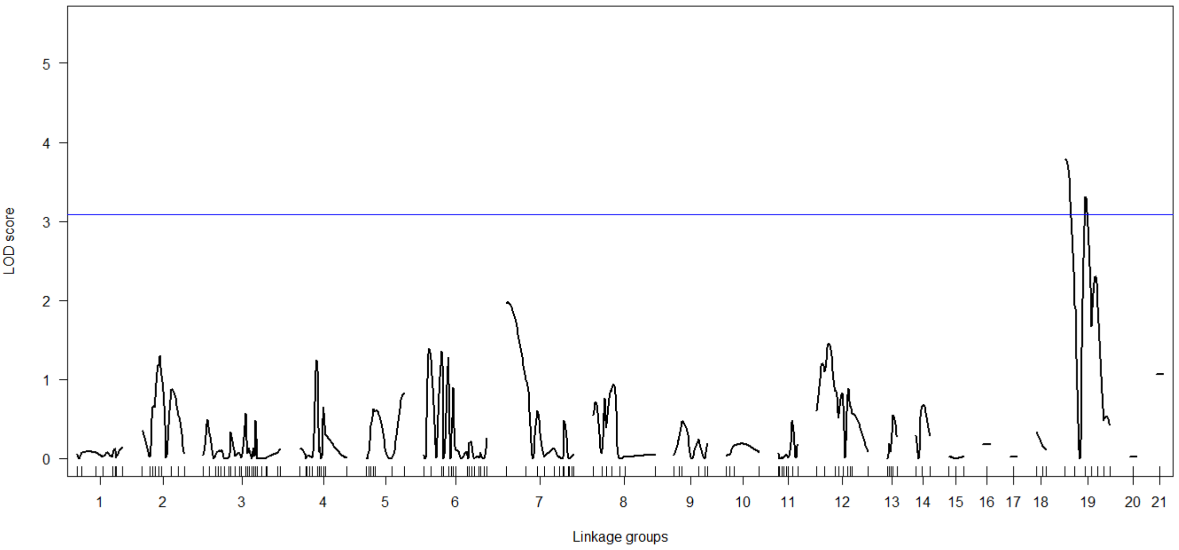

Female A (74F)

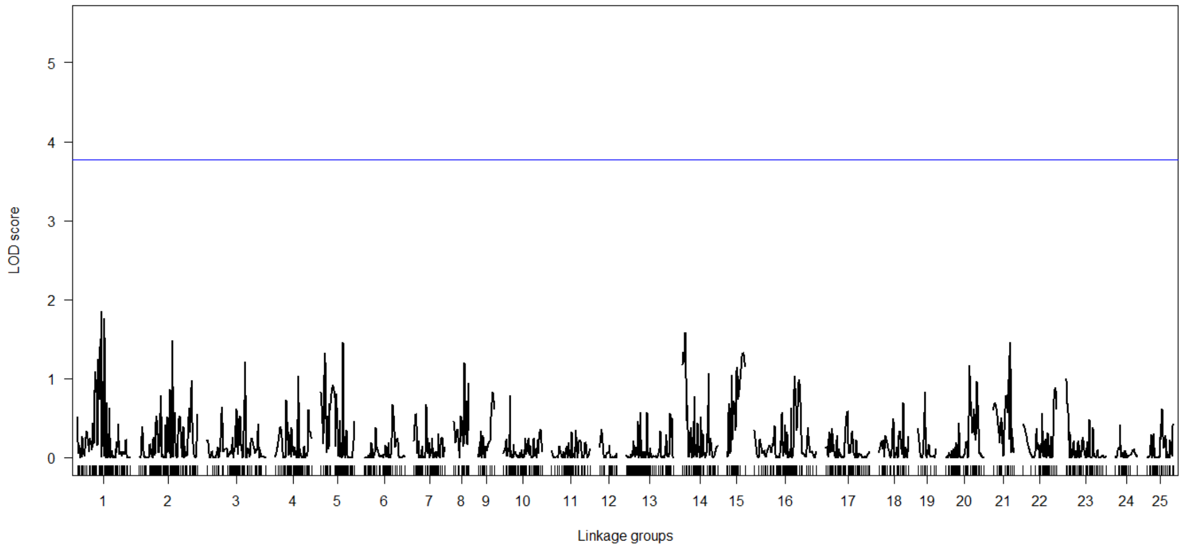

Female B (74F)

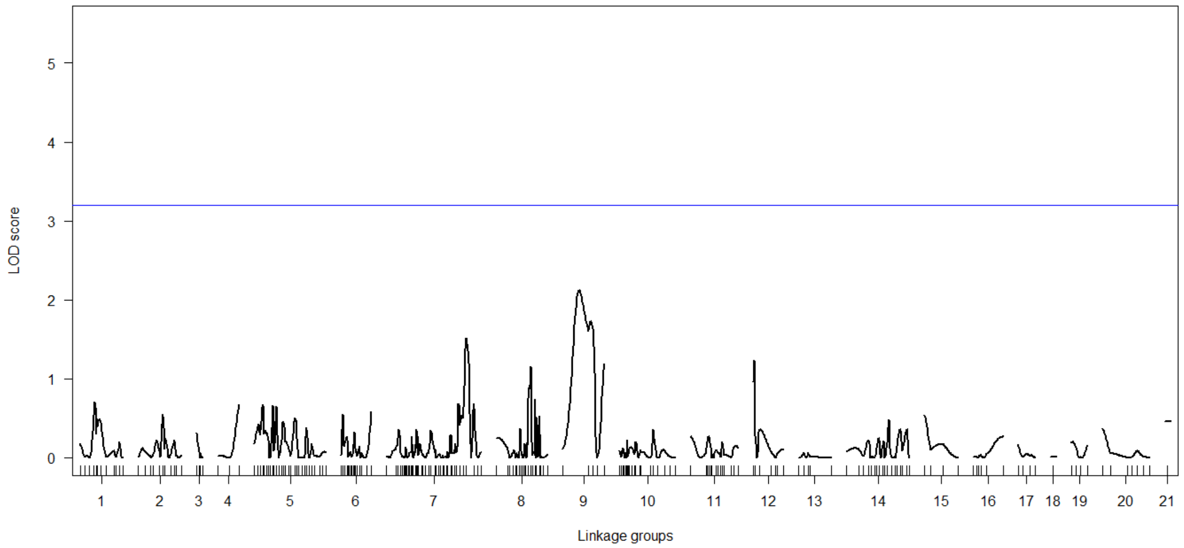

Online resource 6B - Details on significant QTLs: LOD score in function of genetic distance along the sex-LG (LG6_M homologs) by male maps and recombination frequencies heatmaps. LG6_M homologs are LG19 in both male maps (see Online Resource 4). QTL confidence intervals were computed using the “bayesint” function of the R/qtl 1.42-8 package (Broman et al., 2013) with a 0.95 probability coverage of the interval and visualized by a red horizontal lines. Heatmap legend: upper part, recombination frequencies; lower part, LOD.

Male A – Kabusa

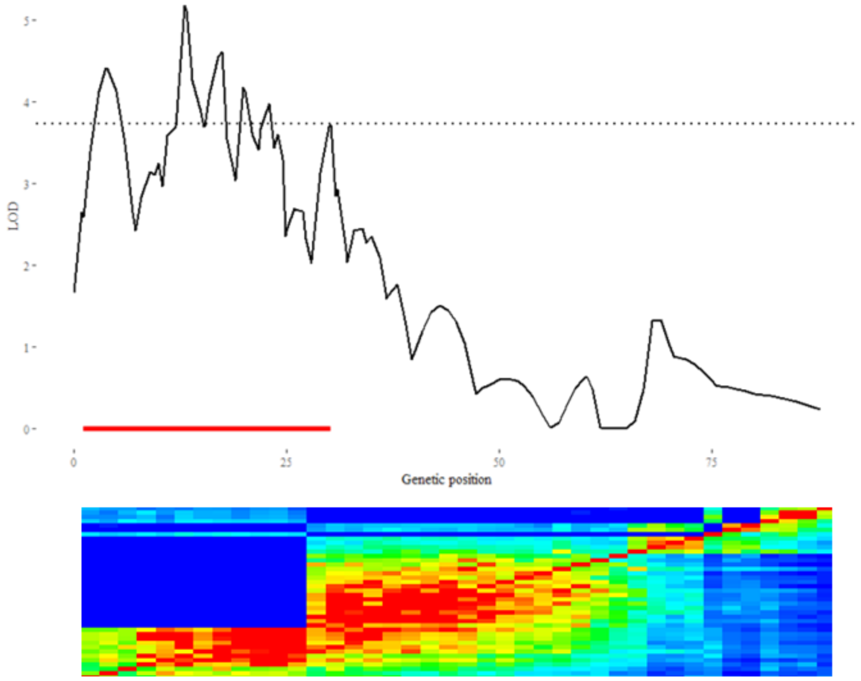

Male B – 14M

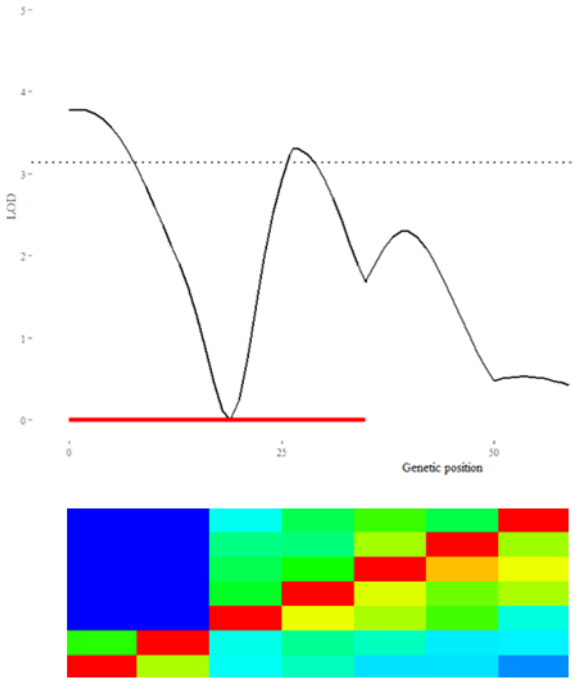

Online resource 7: Distribution of *D. alata* linkage groups (consensus map) within *D. rotundata* linkage groups (% of SNPs).

		D. alata linkage groups																			
		1	2	3	4	5	6_F	6_M	7	8	9	10	11	12	13	14	15	16	17	18	19	20
D. rotundata chromosome	1	0.95	--	--	--	--	--	--	--	--	0.02	--	--	--	--	--	--	--	--	--	--	--
	2	--	0.80	0.02	0.01	--	--	--	0.01	0.01	0.01	0.01	--	0.02	0.02	--	0.01	--	--	0.01	--	0.02
	3	--	0.01	0.94	--	--	--	--	0.01	--	--	--	--	--	--	--	0.02	--	0.01	0.01	--	0.02
	4	--	--	--	0.90	--	--	--	--	--	--	--	0.02	--	0.05	--	0.13	--	--	0.01	--	--
	5	--	--	--	0.03	0.99	--	--	0.02	--	--	0.06	0.02	--	0.03	--	--	--	--	--	0.01	--
	6	--	--	--	0.01	--	0.95	0.94	--	--	--	--	--	0.11	0.06	--	0.12	--	0.01	--	0.03	--
	7	--	0.02	--	0.02	--	0.02	--	0.66	--	--	--	--	--	--	0.05	--	--	0.01	--	--	--
	8	--	0.02	0.01	--	--	--	--	0.05	0.99	0.04	--	--	--	0.02	--	--	--	--	0.01	0.09	0.02
	9	--	0.05	--	--	--	--	--	--	--	0.90	--	--	--	--	--	0.01	--	--	--	0.03	--
	10	--	0.02	--	--	--	--	--	--	--	--	0.89	--	--	0.02	--	--	--	--	--	0.01	--
	11	--	0.01	--	--	--	0.02	0.02	--	--	--	--	0.96	--	--	--	--	--	--	0.01	0.01	--
	12	--	--	0.01	0.01	--	--	--	--	--	--	--	--	0.84	--	--	--	--	--	0.01	--	--
	13	--	--	--	--	--	--	0.02	0.01	--	0.01	0.01	--	--	0.82	--	--	--	0.01	--	0.03	0.02
	14	0.04	0.02	--	--	--	--	--	--	--	--	--	--	0.02	--	0.90	--	--	--	--	--	--
	15	--	--	0.01	--	0.01	--	--	0.23	--	--	0.03	--	--	--	--	0.68	--	--	--	--	--
	16	--	--	--	--	--	--	--	--	--	--	--	--	0.02	--	--	0.01	0.97	--	0.01	0.10	--
	17	--	0.02	--	0.02	--	--	--	--	--	--	--	--	--	--	--	0.01	--	0.94	--	--	--
	18	0.02	0.01	--	--	--	--	--	0.01	--	--	--	--	--	--	0.05	--	--	--	0.92	--	--
	19	--	--	--	--	0.01	--	--	--	--	0.01	--	--	--	--	--	0.02	0.03	0.01	--	0.48	--
	20	--	--	--	--	--	--	0.02	--	--	--	--	--	--	--	--	--	--	--	--	--	0.93
	21	--	0.01	--	--	--	--	--	--	--	--	--	--	--	--	--	--	--	--	--	0.21	--

Online resource 8: Genomic sequence distribution (%) in homolog linkage groups between the reordered *D. rotundata* genome and its published version. (A) In, percentage of the published *D. rotundata* genome (Tamiru *et al*., 2017) (B) in percentage of the *D. rotundata* genome reorder thanks to the consensus *D. alata* genetic map.

(A)

	D. rotundata genome reordered according to the *D. alata* consensus linkage map																				
D. rotundata published by Tamiru *et al.*, 2017	1	2	3	4	5	6_F	6_M	7	8	9	10	11	12	13	14	15	16	17	18	19	20
1	0.85	--	--	--	--	--	--	--	--	0.15	--	--	--	--	--	--	--	--	--	--	--
2	--	0.77	0.03	--	--	--	--	0.03	0.03	0.01	--	--	0.06	0.02	--	0.02	--	--	0.01	--	0.02
3	--	--	0.84	--	--	--	--	0.01	--	--	--	--	--	--	--	0.09	--	0.01	0.01	--	0.04
4	--	--	--	0.89	--	--	--	--	--	--	--	0.01	--	0.01	--	0.08	--	--	0.01	--	--
5	--	--	--	0.05	0.86	--	--	0.05	--	--	0.03	0.01	--	0.01	--	--	--	--	--	--	--
6	--	--	--	--	--	0.51	0.61	--	--	--	--	--	0.04	0.02	--	0.15	--	0.01	--	0.16	--
7	--	0.01	--	0.06	--	0.12	--	0.73	--	--	--	--	--	--	0.06	--	--	0.03	--	--	--
8	--	0.06	--	--	--	--	--	0.05	0.69	0.02	--	--	--	0.02	--	--	--	--	0.05	0.08	0.03
9	--	0.02	--	--	--	--	--	--	--	0.91	--	--	--	--	--	0.05	--	--	--	0.02	--
10	--	0.05	--	--	--	--	--	--	--	--	0.92	--	--	0.02	--	--	--	--	--	0.01	--
11	--	0.02	--	--	--	0.03	--	--	--	--	--	0.90	--	--	--	--	--	--	0.03	0.02	--
12	--	--	0.04	0.04	--	--	--	--	--	--	--	--	0.73	--	--	--	--	--	0.19	--	--
13	--	--	--	--	--	--	0.01	0.02	--	0.02	0.02	--	--	0.75	--	--	--	0.03	--	0.13	0.02
14	0.08	0.21	--	--	--	--	--	--	--	--	--	--	0.09	--	0.63	--	--	--	--	--	--
15	--	--	0.01	--	0.02	--	--	0.17	--	--	0.03	--	--	--	--	0.77	--	--	--	--	--
16	--	--	--	--	--	--	--	--	--	--	--	--	0.25	--	--	--	0.12	--	--	0.63	--
17	--	0.01	--	0.09	--	--	--	--	--	--	--	--	--	--	--	--	--	0.89	--	--	--
18	--	--	--	--	--	--	--	0.01	--	--	--	--	--	--	0.04	--	--	--	0.95	--	--
19	--	--	--	--	--	--	--	--	--	0.04	--	--	--	--	--	0.02	0.04	0.08	--	0.81	--
20	--	--	--	--	--	--	0.05	--	--	--	--	--	--	--	--	--	--	--	--	--	0.95
21	--	0.10	--	--	--	--	--	--	--	--	--	--	--	--	--	--	--	--	--	0.90	--

(B)

		D. rotundata genome reordered according to the *D. alata* consensus linkage map																			
		1	2	3	4	5	6_F	6_M	7	8	9	10	11	12	13	14	15	16	17	18	19	20
D. rotundata published by Tamiru *et al.*, 2017	1	0.96	--	--	--	--	--	--	--	--	0.17	--	--	--	--	--	--	--	--	--	--	--
	2	--	0.77	0.06	--	--	--	--	0.05	0.04	0.01	--	--	0.07	0.03	--	0.03	--	--	0.02	--	0.05
	3	--	--	0.88	--	--	--	--	0.01	--	--	--	--	--	--	--	0.09	--	0.01	0.01	--	0.06
	4	--	--	--	0.82	--	--	--	--	--	--	--	0.02	--	0.02	--	0.11	--	--	0.01	--	--
	5	--	--	--	0.05	0.99	--	--	0.08	--	--	0.05	0.01	--	0.01	--	--	--	--	--	--	--
	6	--	--	--	--	--	0.86	0.95	--	--	--	--	--	0.05	0.02	--	0.26	--	0.02	--	0.13	--
	7	--	--	--	0.03	--	0.11	--	0.67	--	--	--	--	--	--	0.09	--	--	0.03	--	--	--
	8	--	0.05	--	--	--	--	--	0.07	0.96	0.02	--	--	--	0.02	--	--	--	--	0.04	0.06	0.06
	9	--	0.02	--	--	--	--	--	--	--	0.76	--	--	--	--	--	0.07	--	--	--	0.01	--
	10	--	0.03	--	--	--	--	--	--	--	--	0.90	--	--	0.01	--	--	--	--	--	0.01	--
	11	--	0.01	--	--	--	0.03	--	--	--	--	--	0.96	--	--	--	--	--	--	0.02	0.01	--
	12	--	--	0.06	0.03	--	--	--	--	--	--	--	--	0.63	--	--	--	--	--	0.17	--	--
	13	--	--	--	--	--	--	0.02	0.03	--	0.02	0.03	--	--	0.89	--	--	--	0.04	--	0.10	0.05
	14	0.04	0.10	--	--	--	--	--	--	--	--	--	--	0.05	--	0.83	--	--	--	--	--	--
	15	--	--	--	--	0.01	--	--	0.09	--	--	0.02	--	--	--	--	0.43	--	--	--	--	--
	16	--	--	--	--	--	--	--	--	--	--	--	--	0.20	--	--	--	0.87	--	--	0.37	--
	17	--	0.01	--	0.06	--	--	--	--	--	--	--	--	--	--	--	--	--	0.87	--	--	--
	18	--	--	--	--	--	--	--	0.01	--	--	--	--	--	--	0.07	--	--	--	0.74	--	--
	19	--	--	--	--	--	--	--	--	--	0.02	--	--	--	--	--	0.01	0.13	0.04	--	0.20	--
	20	--	--	--	--	--	--	0.03	--	--	--	--	--	--	--	--	--	--	--	--	--	0.78
	21	--	0.02	--	--	--	--	--	--	--	--	--	--	--	--	--	--	--	--	--	0.12	--

Online resource 9: Genetic position in the consensus map versus physical position in the *D. rotundata* genome reordered using the consensus *D. alata* map.

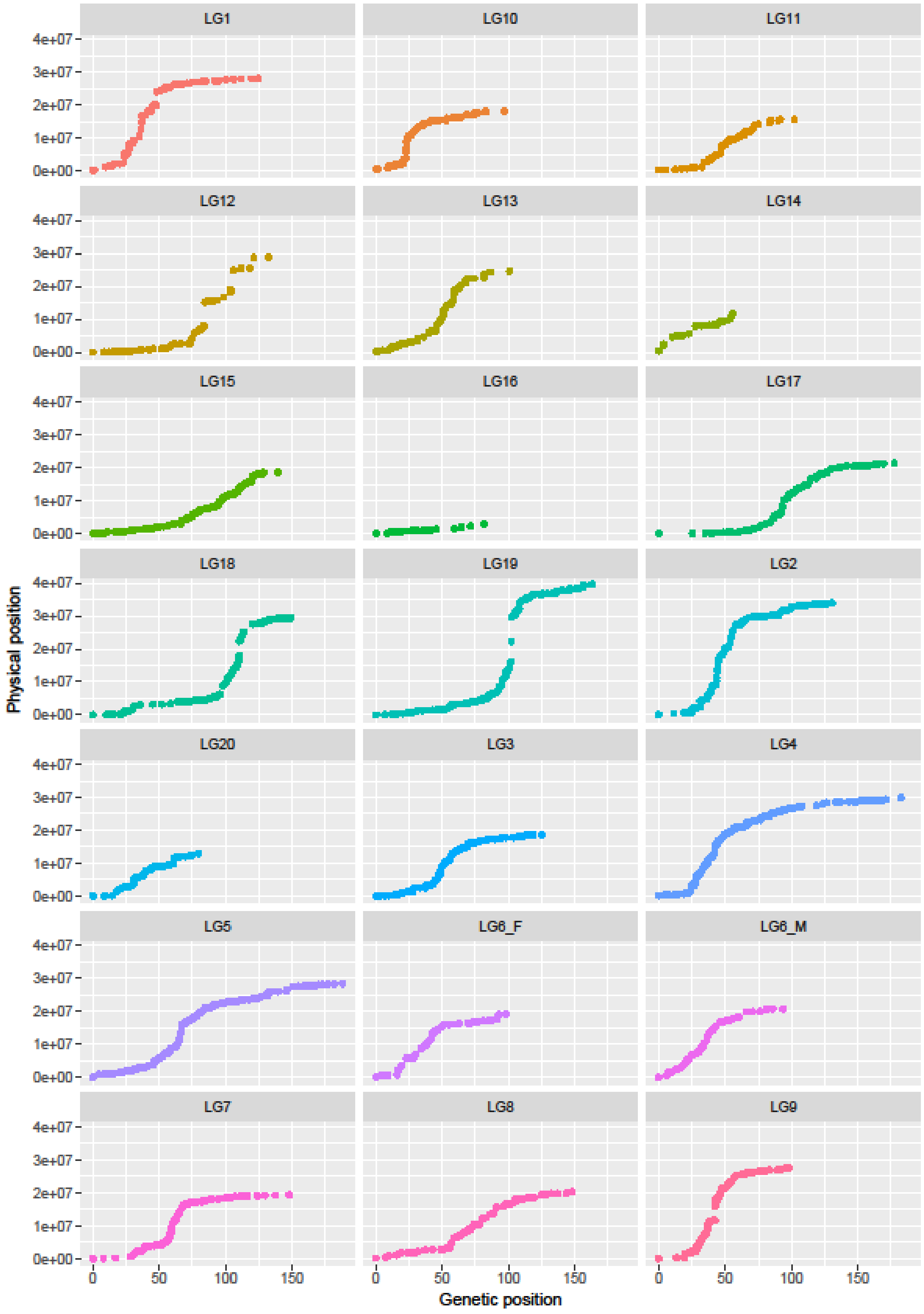

Online resource 11: Physical distance between SNPs in *D. alata* scaffolds in function of the physical distance in the *D. rotundata* reference genome. Distance between pairs of SNPs located in the same *D. alata* scaffold and *D. rotundata* chromosome and spaced less than 1 Mb (213 scaffolds; 1 178 SNPs pairs; distance in *D. alata* = 1.12e+04 + distance in *D. rotundata* × 0.786; $r^2 = 0.79$).

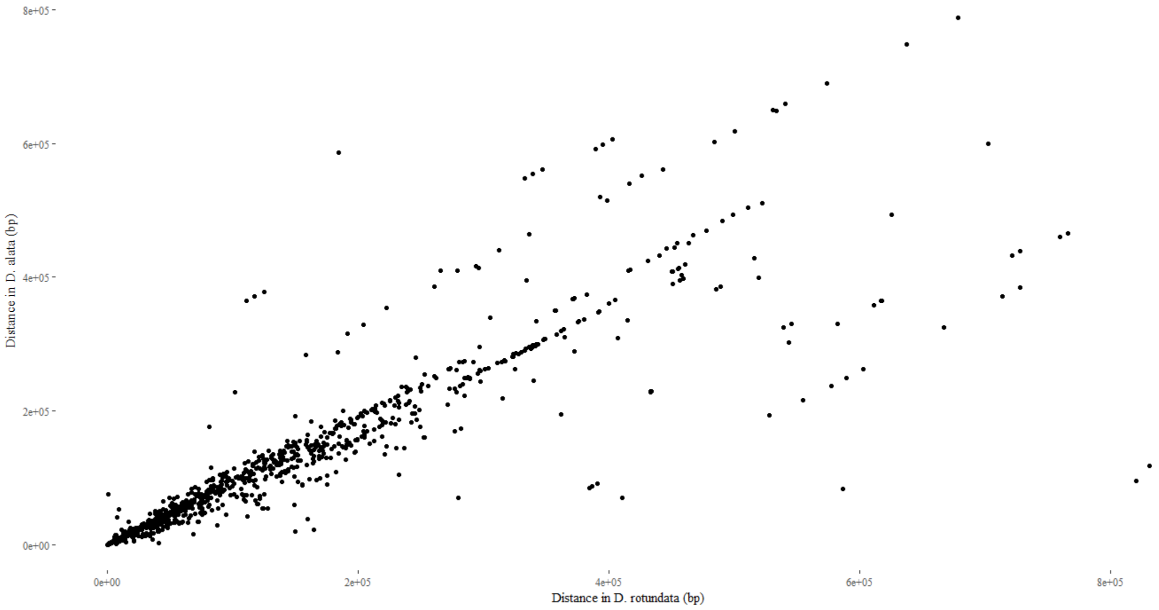

Supplement: Supplementary file 1 — Supplementary material 1 (PDF 3121 kb) [file 122_2019_3311_MOESM1_ESM.pdf]
